# Complex interplay of neurodevelopmental disorders (NDDs), fractures, and osteoporosis: a mendelian randomization study

**DOI:** 10.1186/s12888-024-05693-4

**Published:** 2024-03-27

**Authors:** Zefang Li, Xueqiang Wu, Hanzheng Li, Cong Bi, Can Zhang, Yiqing Sun, Zhaojun Yan

**Affiliations:** 1 Department of The First Clinical medicine, Shandong University of Traditional Chinese Medicine, Jinan, China; 2 Department of Health Science, Shandong University of Traditional Chinese Medicine, Jinan, China; 3 Department of Vascular Surgery, Shandong Provincial Hospital Affiliated to Shandong First Medical University, Jinan, China; 4 School of Biomedical Sciences, Shandong First Medical University, Jinan, China; 5 Affiliated Hospital of Shandong University of Traditional Chinese Medicine, Jinan, China

**Keywords:** Neurodevelopmental disorders, Attention deficit hyperactivity disorder, Autism spectrum disorder, Tourette syndrome, Mendelian randomization, Fracture and osteoporosis

## Abstract

**Background:**

Neurodevelopmental disorders (NDDs), such as Attention-Deficit/Hyperactivity Disorder (ADHD), Autism Spectrum Disorder (ASD), and Tourette Syndrome (TS), have been extensively studied for their multifaceted impacts on social and emotional well-being. Recently, there has been growing interest in their potential relationship with fracture risks in adulthood. This study aims to explore the associations between these disorders and fracture rates, in order to facilitate better prevention and treatment.

**Methods:**

Employing a novel approach, this study utilized Mendelian randomization (MR) analysis to investigate the complex interplay between ADHD, ASD, TS, and fractures. The MR framework, leveraging extensive genomic datasets, facilitated a systematic examination of potential causal relationships and genetic predispositions.

**Results:**

The findings unveil intriguing bidirectional causal links between ADHD, ASD, and specific types of fractures. Notably, ADHD is identified as a risk factor for fractures, with pronounced associations in various anatomical regions, including the skull, trunk, and lower limbs. Conversely, individuals with specific fractures, notably those affecting the femur and lumbar spine, exhibit an increased genetic predisposition to ADHD and ASD. In this research, no correlation was found between TS and fractures, or osteoporosis.These results provide a genetic perspective on the complex relationships between NDDs and fractures, emphasizing the importance of early diagnosis, intervention, and a holistic approach to healthcare.

**Conclusion:**

This research sheds new light on the intricate connections between NDDs and fractures, offering valuable insights into potential risk factors and causal links. The bidirectional causal relationships between ADHD, ASD, and specific fractures highlight the need for comprehensive clinical approaches that consider both NDDs and physical well-being.

**Supplementary Information:**

The online version contains supplementary material available at 10.1186/s12888-024-05693-4.

## Introduction

Attention Deficit Hyperactivity Disorder (ADHD), Autism Spectrum Disorder (ASD), and Tourette Syndrome (TS) are prominent Neurodevelopmental disorders(NDDs)often co-occurring and impactingsocial and emotional aspects [1]. Observational studies reveal higher fracture risks among adults with these conditions compared to those without [2]. Notably, ADHD is associated with an increased risk of fractures, highlighting the importance of recognizing the elevated risk in individuals with ADHD [3–6].

The core features of ADHD involve attention deficits, heightened hyperactivity, and impulsive behaviors. Notably, children with ADHD face an elevated risk of fractures, particularly affecting the skull, neck, trunk, and intracranial structures, with a Hazard Ratio (HR) 3.07 times higher than neurotypical peers [7]. Research highlights the statistical significance (*P* < 0.001) of this heightened fracture risk, encompassing regions such as the skull, neck, and trunk (HR = 1.53), upper limbs (HR = 1.28), and lower extremities (HR = 1.84) [5]. Moreover, studies indicate a correlation between ADHD symptoms and limb fractures in adults [8], with a notable reduction in fracture risk for individuals receiving pharmacological interventions [9, 10].

ASD is a NDDs by social impairment and restricted interactive and communicative behaviors [11]. Notably, individuals within the ASD spectrum exhibit an increased susceptibility to injuries involving the head, face, and neck [12], as well as a heightened risk of fractures affecting the hip, forearm, and spine [13]. Additionally, adolescent boys with ASD display lower bone mineral density (BMD) in comparison to their neurotypical peers [14].

Research consistently reveals distinctive patterns in bone health among individuals with ASD, including reduced systemic bone mineral content, diminished cortical area, and thinning of cortical and trabecular bone structures, especially in the distal radius and tibia. These trends extend to various anatomical regions, such as the lumbar spine, hips, and femoral neck, setting individuals with ASD apart from those without the condition [15].

TS is an intricate NDDs characterized by the presence of persistent vocal and motor tics, a defining feature that must endure for at least one year. Notably, a comprehensive decade-long cohort study unveiled a 1.27-fold higher fracture rate in individuals within the TS cohort when compared to a meticulously matched control cohort. Furthermore, a thorough multivariate Cox regression analysis revealed an adjusted HR of 1.28. Of particular interest is the observation that the introduction of antipsychotic medications led to a notable reduction in this heightened risk, resulting in a decreased HR of 1.17 [16]. This phenomenon warrants investigation, considering factors such as self-harm, accidental injuries, and the potential consequences of antipsychotic medication-related side effects [16, 17].

Fractures and osteoporosis cast a significant shadow over public health [18], with a disquieting projection of over 30 million individuals expected to be affected by bone-related conditions in Europe by the year 2050. The associated financial burden is equally disconcerting, with potential hospitalization costs in Europe alone estimated to reach up to €3.5 billion annually [19]. It is crucial to acknowledge that while genetics play a substantial role, one’s bone structure is subject to modification, influenced by intrinsic and extrinsic factors such as physical activity, hormonal dynamics, and nutritional status [20].

The pivotal role of nutrition and physical activity takes center stage in strengthening bones during crucial early developmental stages. This emphasis on early intervention stems from the understanding that as individuals progress through life, the gradual accrual of undesirable bone mass can lead to bone fragility and, ultimately, the onset of osteoporosis. Aligned with the insights of the International Osteoporosis Foundation, osteoporosis is aptly defined as a condition characterized by low bone mass and the progressive deterioration of bone microstructure, culminating in heightened bone fragility and an elevated risk of fractures [21].

Fractures in the Pediatric and Adolescent Realm: A Significant Component of Injuries:

Fractures hold a significant place in the realm of accidents and injuries, accounting for a substantial 25% of all such incidents involving children and adolescents. While a wide array of risk factors has been identified, including factors like nutritional deficiencies [22], high body weight, and intense physical activities [23], as well as psychiatric disorders such as schizophrenia [24], the intricate interplay between fractures, osteoporosis, and the three NDDs of ADHD, ASD, and TS warrants comprehensive examination.

Simultaneously, cross-disease investigations have uncovered significant overlap in the phenotypic traits associated with the genetic risk shared by ADHD, ASD, and TS [1]. Grounded in the characteristic features of inattention, hyperactivity, and impulsivity commonly observed in individuals with ADHD, previous research provides a compelling basis for postulating causal links between ADHD, ASD, TS and the occurrence of fractures and osteoporosis. Furthermore, considering the associations among ADHD, ASD, TS, and intellectual disabilities, all of which fall within the spectrum of NDDs [25, 26], we have deliberately incorporated intellectual functioning into the scope of our investigation to enhance the credibility of our research. It is essential to recognize that these causal relationships may exhibit distinct patterns across various anatomical sites.

Mendelian randomization (MR) methodologies harness the potential of single nucleotide polymorphisms (SNPs) as instrumental variables(IVs) to rigorously estimate the causal relationships between exposures and specific outcomes. This approach offers a distinct advantage by significantly mitigating the impact of confounding variables and measurement errors that often confound traditional multivariate regression models [27]. Moreover, the deliberate exclusion of IVs that overlap with confounding factors in MR analyses minimizes the risk of bias, thereby enhancing the validity of causal inferences [28].

Our primary aim is to utilize the robust MR methodology for a comprehensive evaluation of the causal associations between ADHD, ASD, TS and an elevated genetic predisposition to fractures in diverse anatomical regions.

## Methods

### Data source for NDDs

The genomic association data for ADHD [29], ASD [30], TS [31] utilized in this study were extracted from the PGC database. The genomic association data for intelligence [32] were extracted from Complex Trait Genetics Lab (CTG) (https://ctg.cncr.nl/).

The Population Characteristics:

All participants included in the study were of European descent. The diagnostic criteria for ADHD and ASD were based on the ICD-10 (International Classification of Diseases, 10th Revision), while the diagnostic criteria for Tourette Disorder adhered to the DSM-5 (Diagnostic and Statistical Manual of Mental Disorders, Fifth Edition).

Instrumental Variables (IVs):

The IVs employed in the analysis consisted of a total of 582 SNPs. These SNPs were meticulously selected based on specific criteria, including a significance threshold of *p* < 5 × 10^-6, minimal linkage disequilibrium (LD) interlocking imbalance (LD r^2 < 0.001), and a clumping distance exceeding 10,000 kilobases. Detailed information about these IVs can be found in Supplementary Table.

### Data source for fractures and osteoporosis

The data related to fractures and osteoporosis in this study were sourced from the FinnGen project [33], specifically utilizing the R9 version of the data, which was published on May 11, 2023. Detailed instructions and information about this research project and its data sources are available on the project’s website: FinnGen Project Information.

Diagnostic Criteria and Participant Characteristics:

The diagnosis of fractures and osteoporosis across these categories was based on the ICD-10. All individuals included in the study were of European descent.

Instrumental Variables (IVs):

IVs comprised a total of 269 SNPs. These SNPs were carefully selected based on specific criteria, including a significance threshold of *p* < 5 × 10^-6, minimal LD interlocking imbalance (LD r^2 < 0.001), and a clumping distance exceeding 10,000 kilobases. Comprehensive information regarding these IVs can be found in Supplementary Table.

**The Details of cohort specifics of NDDs and Fractures and Osteoporosis are presented in** Table 1.

**Table 1:** Detailed information of the datasets used for MR analyses

Traits	Year	Authors	Population	Consortium	Sample size	Number of SNPs	
ADHD	2023	Havdahl et al.	European	PGC	225,534	6,774,224	
ASD	2019	Grove et al.	European	PGC	43,350	9,112,386	
TS	2019	Yu et al.	European	PGC	14,307	8,265,318	
Intelligence	2018	Savage et al.	European	CTG	269,867	9,295,118	
Fracture of femur	2023	Kurki et al.	European	Finngen	369,694	20,170,071	
Osteoporosis	2023	Kurki et al.	European	Finngen	365,314	20,169,923	
Osteoporosis with pathological fracture (FG)	2023	Kurki et al.	European	Finngen	286,694	20,167,428	
Fracture of shoulder and upper arm	2023	Kurki et al.	European	Finngen	356,923	20,169,771	
Fracture of neck	2023	Kurki et al.	European	Finngen	371,696	20,170,126	
Fracture of rib(s), sternum, and thoracic spine	2023	Kurki et al.	European	Finngen	371,354	20,170,108	
Fracture of skull and facial bones	2023	Kurki et al.	European	Finngen	336,225	20,169,238	
Fracture of forearm	2023	Kurki et al.	European	Finngen	370,773	20,170,116	
Fracture at the wrist and hand level	2023	Kurki et al.	European	Finngen	347,812	20,169,593	
Fracture of the lower leg (including ankle)	2023	Kurki et al.	European	Finngen	343,301	20,169,406	
Fracture of lumbar spine and pelvis	2023	Kurki et al.	European	Finngen	370,713	20,170,079	
Fracture of the foot (except ankle)	2023	Kurki et al.	European	Finngen	358,985	20,169,888	

### Confounding factors

Numerous studies have highlighted a significant connection between early life-related traits and the emergence of neurological and psychiatric disorders in adulthood, potentially due to increased oxidative stress within the central nervous system during early life [34]. For example, a well-documented association exists between low birth weight and ADHD [35]. Additionally, the mounting prevalence of childhood obesity has emerged as a significant public health concern due to its intricate and multifaceted relationship with neurodevelopmental health challenges in adulthood [36]. Furthermore, it is worth mentioning that allergic diseases during childhood hold the potential to precipitate ADHD symptoms [37].

Management of Confounding Factors:

Moreover, existing literature has indicated that the intake of vitamin D [38], calcium [39], and zinc [40] serve as risk factors for fractures and osteoporosis. Supplementation with vitamin D has been shown to augment bone density, thereby mitigating the risks of fractures and osteoporosis [38]. Calcium and vitamin D have been identified as pivotal determinants of peak bone mass [39]. Conversely, a diminished intake of zinc is associated with an elevated risk of fractures and osteoporosis [40]. Consequently, we have incorporated dietary supplements of vitamin D, calcium, and zinc as confounding variables in this study.

In light of the aforementioned considerations, this study rigorously controlled for potential confounding factors. It incorporated birth height [41], birth weight [42], body mass index (BMI) [43], and the presence of allergic diseases within the analytical framework. The impact of these factors on the observed outcomes was meticulously addressed by excluding SNPs that showed overlap within the IVs. Birth height, weight, and children’s BMI data were sourced from The Early Growth Genetics (EGG) Consortium, while information on allergic diseases was acquired from a Finnish database [33]. The data on theintakeof vitamin D, calcium, and zinc were sourced from the IEU Open GWAS database [44]. This comprehensive approach enhances the study’s robustness, minimizing the influence of potential confounders and bolstering the precision of causal inferences.

### Statistical analysis

This study employed a MR approach to explore the intricate bidirectional relationship between ADHD, ASD, TS, and 11 distinct types of fractures and osteoporosis.

**Fig. 1:**
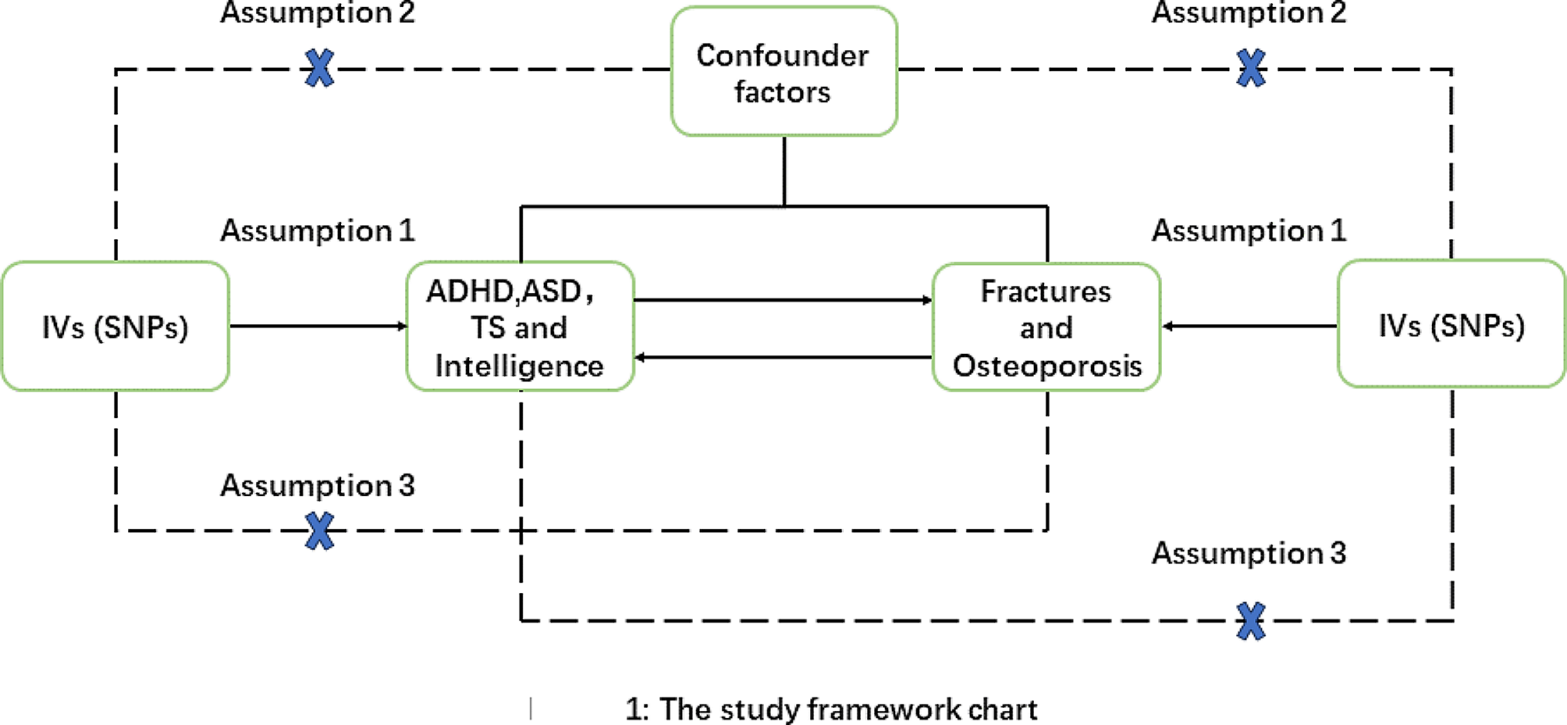
MR assumptions and design of our study:1. IV Exposure Association: IVs demonstrated a robust association with the exposure of interest.2. Absence of Unidentified Confounding: IVs were selected to have no associations with unidentified confounding factors that could affect both the exposure and outcome.3. IV Effect via Exposure: IVs exclusively influenced the outcomes through the exposure under investigation

The study adhered to the classical MR assumptions (Fig. 1)

To mitigate confounding factors, the study rigorously screened IVs and established a significance threshold (*P* < 5e-06) to minimize weak associations between potential confounders and genetic variations.

The primary statistical metric used was the inverse variance weighted (IVW) method, assessing the association between ADHD, ASD, and fractures or osteoporosis. MR-Egger, weighted median, and weighted models served as supplementary statistical indicators.

Sensitivity analysis, a crucial component of MR analysis, was employed to detect potential pleiotropy. Heterogeneity was assessed using the Cochrane Q test, and horizontal pleiotropy was examined via the MR-Egger intercept method. A leave-one-out analysis assessed the impact of individual SNPs on MR results. In cases of heterogeneity, the random-effect IVW was used as the primary statistical measure, while the fixed-effect IVW was employed when no heterogeneity was observed.

The significance threshold for results was corrected for multiple testing using the Bonferroni method. A p-value of < 0.001 (0.05/36) was considered significant, p-values between 0.001 and 0.05 were considered suggestive, and p-values above 0.05 were deemed insignificant. The F statistic $(F=\frac{bet{a}^{2}}{s{e}^{2}})$ was used to assess the strength of IVs, with F > 10 indicating a robust and independent instrumental variable [45].

All statistical analyses were performed using the TwoSample MR package in R version 4.2.2, and the results were reported in terms of odds ratio (OR) values with associated 95% confidence intervals.

## Result

### Causal relationship between fractures and osteoporosis and ADHD, ASD, TS and intelligence

**Fig. 2:**
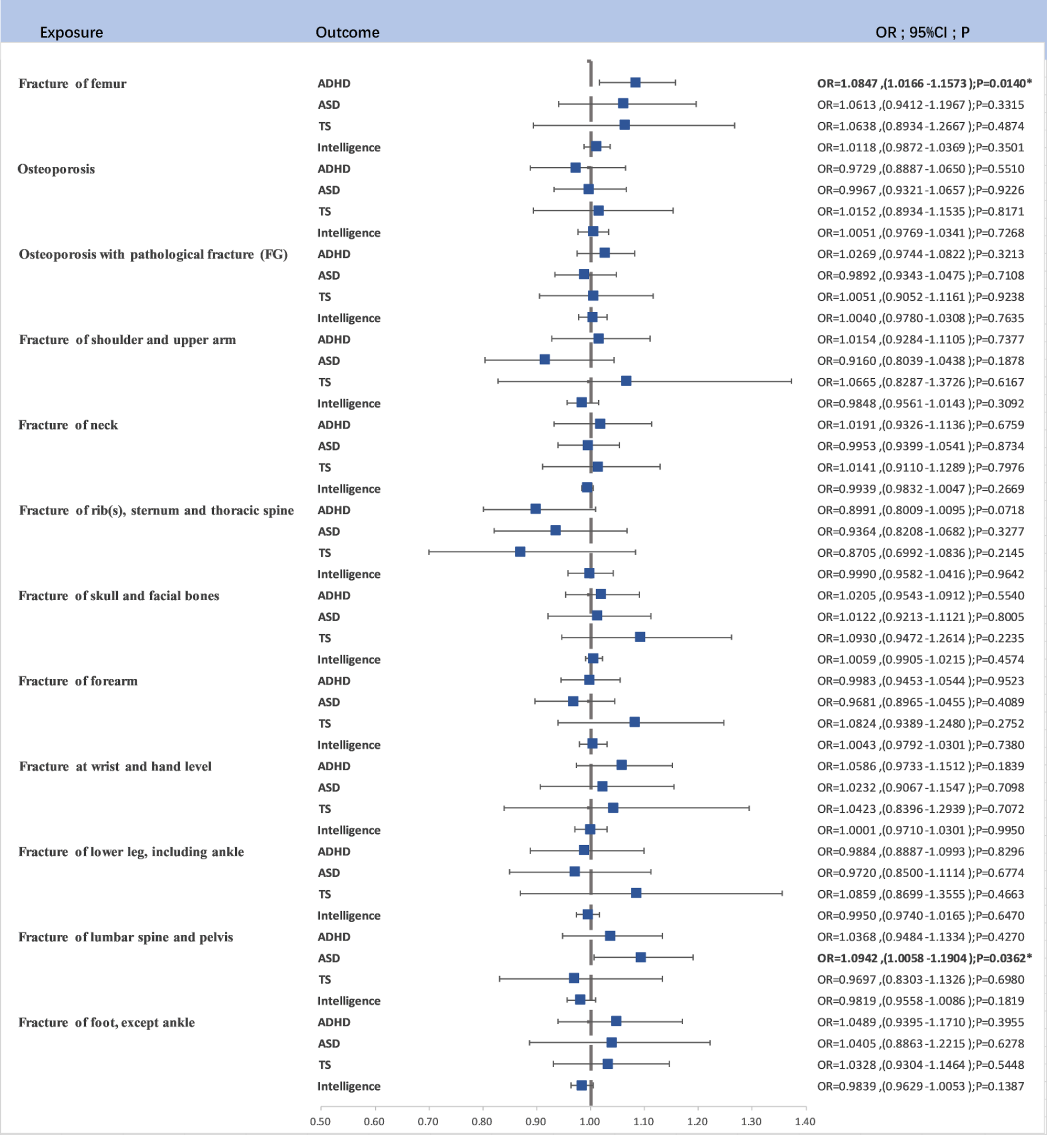
The forest map for Causal Relationship Between Fractures and Osteoporosis and ADHD, ASD, and TS;*: 0.05 < *P* < 0.001, ***P < 0.001

A similar screening process, accounting for P-values and LD clumping, was applied to SNPs used as IVs for different fracture and osteoporosis outcomes. Although potential causal relationships were detected, they did not consistently reach statistical significance after Bonferroni correction (Fig. 2)

Fracture of femur and ADHD: A suggestive causal relationship was observed (OR = 1.0847, 95% CI (1.0166–1.1573), *P* = 0.0140).

Fracture of lumbar spine and pelvis and ASD: Another suggestive causal relationship was noted (OR = 1.0942, 95% CI (1.0058–1.1904), *P* = 0.0362).

These findings, while suggestive, did not maintain statistical significance following Bonferroni correction but offer valuable insights into potential risk factors.

Heterogeneity and Pleiotropy Testing:

Detailed information on the results of heterogeneity and pleiotropy testing is available in the supplementary material and supplementary table, providing a comprehensive assessment of the findings’ robustness in light of these factors.

### Causal relationship between ADHD, ASD, TS, intelligence and fracture risk

**Fig. 3:**
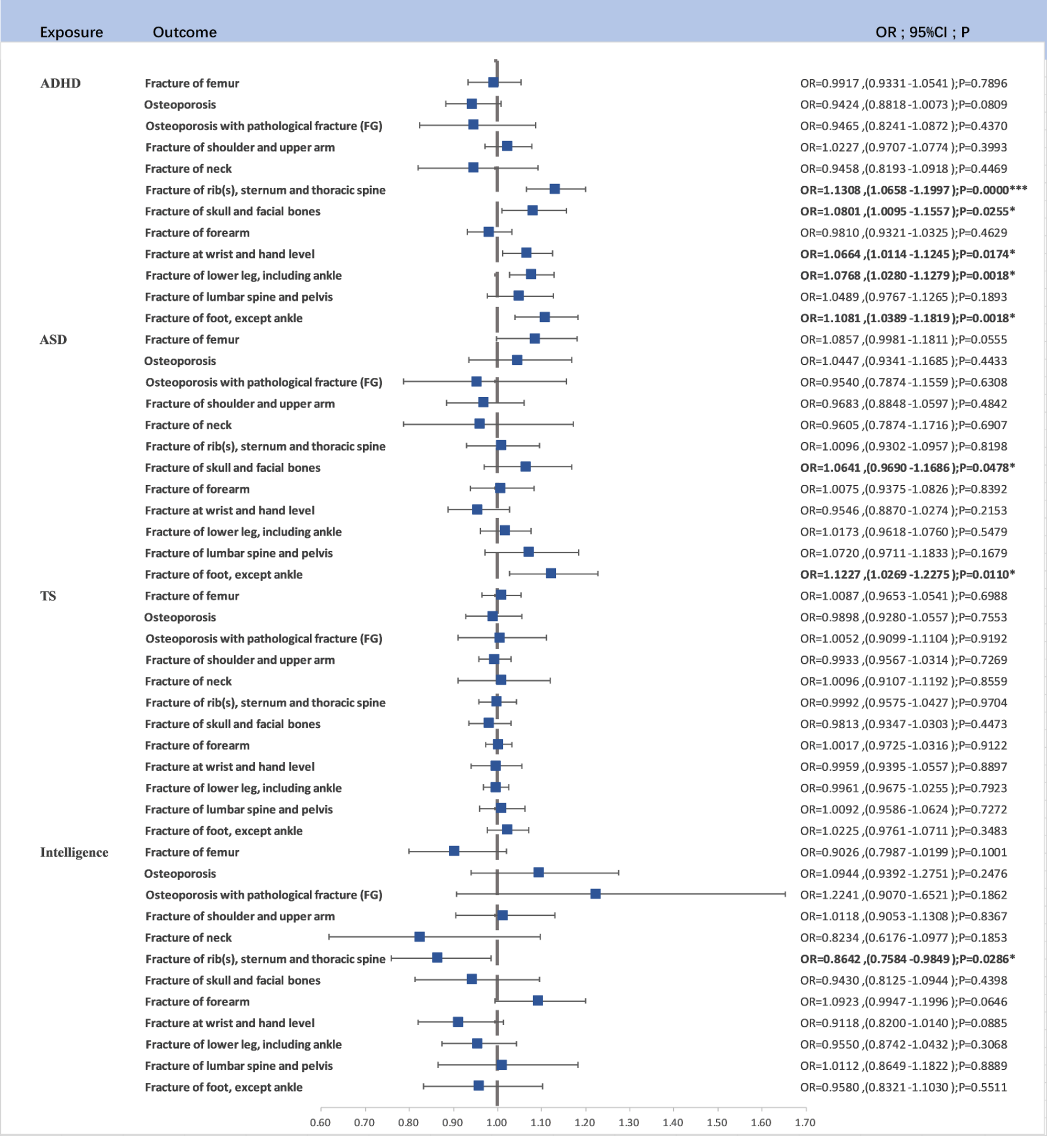
The forest map for Causal Relationship Between ADHD, ASD, TS, and Fracture Risk*: 0.05 < *P* < 0.001, ***P < 0.001

After rigorous SNP screening based on P-values and LD clumping, a total of 138, 34, 22 and 387 linkage-disequilibrium-independent SNPs served as IVs for ADHD, ASD, TS and Intelligence. Upon eliminating SNPs not present in the outcome variable, a two-sample MR analysis unveiled significant findings (Fig. 3).

ADHD as a Risk Factor: ADHD was identified as a risk factor for Fracture of rib(s), sternum, and thoracic spine (OR = 1.1308, 95% CI (1.0658–1.1997), *P* < 0.0001). This association retained significance after Bonferroni correction. ADHD was also linked to Fracture of foot (except ankle) (OR = 1.1081, 95% CI (1.0389–1.1819), *P* = 0.0018), Fracture of lower leg (including ankle) (OR = 1.0768, 95% CI (1.0280–1.1279), *P* = 0.0018), Fracture at wrist and hand level (OR = 1.0664, 95% CI (1.0114–1.1245), *P* = 0.0174), and Fracture of skull and facial bones (OR = 1.0801, 95% CI (1.0095–1.1557), *P* = 0.0255).

ASD as a Risk Factor: ASD was found to be associated with an increased risk of Fracture of foot (except ankle) (OR = 1.1227, 95% CI (1.0269–1.2275), *P* = 0.0110).

TS as a Risk Factor: No significant causal relationship wasfoundin TS.

Additionally, we observed that higher levels of intelligence were associated with a reduced risk of fractures involving the rib(s), sternum, and thoracic spine (OR = 0.8642, 95% CI (0.7584–0.9849), *P* = 0.0286). The pleiotropy and heterogeneity test results are included in the supplementary materials.

## Discussion

**Fig. 4:**
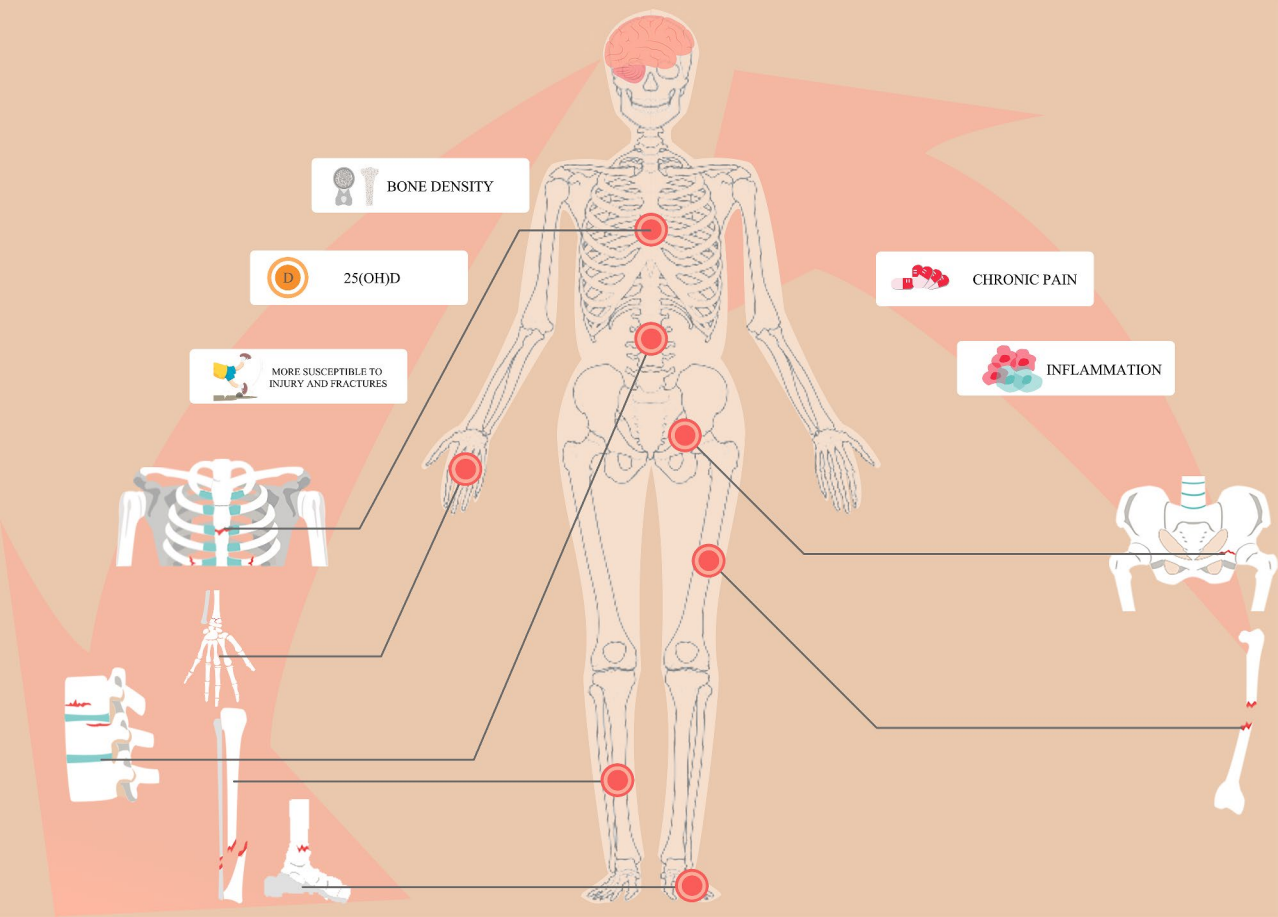
The Potential mechanisms for our study

This comprehensive study adopted a bidirectional MR approach to investigate the intricate relationships between three NDDs—ADHD, ASD, and TS—and a spectrum of 11 distinct fractures and osteoporosis. The MR analysis provided intriguing insights into these associations, shedding light on potential causal links and risk factors (Fig. 4).

### Fractures as causal factors

The MR analysis results suggested that certain fractures may act as causal factors in the development of these childhood NDDs. Specifically, a fracture of the femur may be a risk factor for ADHD (*P* = 0.0140), and fractures of the lumbar spine and pelvis may be risk factors for ASD (*P* = 0.0362). Although these findings did not retain statistical significance after Bonferroni correction, they offer valuable indications of potential associations.

### NDDs as causal factors

Conversely, the reverse MR analysis presented robust findings indicating that ADHD could be a causal factor for various types of fractures. For example, ADHD is a risk factor for fractures of the rib(s), sternum, and thoracic spine (*P* < 0.0001), fractures of the foot (except ankle) (*P* = 0.0018), fractures of the lower leg (including ankle) (*P* = 0.0003), fractures at the wrist and hand level (*P* = 0.0174), and fractures of the craniofacial region (*P* = 0.0255). Similarly, ASD is considered a risk factor for foot fractures (*P* = 0.0110), and higher intelligence levels are associated with a preventive effect against the occurrence of fractures involving the rib(s), sternum, and thoracic spine (*P* = 0.0286). Besides, there is no causal relationship between fractures and TS.

Observational studies have historically grappled with the intricate connections among ADHD, ASD, TS, and fractures, as well as osteoporosis. Often, symptoms linked to these NDDs may unintentionally escape attention when investigating fractures [46]. Our study represents a pioneering effort, constituting the inaugural systematic exploration of the link between childhood NDDs and fractures and osteoporosis through a genetic framework. By adeptly sidestepping potential confounding variables, measurement inaccuracies, and other sources of bias, the study yields invaluable genetic insights. Significantly, it identifies NDDs like ADHD as potential contributors to various fractures, while also suggesting that fractures might be potential causes of diverse fractures. However, this perspective requires further research for clarification. It introduces a new perspective on the potential interplay between psychological well-being and bone health.

Fractures not only induce pain and inflammatory reactions but recent research has also uncovered connections between NDDs and pain [47, 48]. Interestingly, the prevalence of ADHD is significantly higher in adolescents experiencing chronic pain [49–51], and inflammation is suggested as a contributing factor in the development of ADHD and ASD [52, 53]. For instance, inflammatory pain in newborns and responses in preterm infants may elevate the risk of NDDs [54]. In individuals with ADHD, elevated levels of inflammatory biomarkers and inflammation within the central nervous system are believed to contribute to pain perception and sensitization [54, 55]. This also holds promise for preventing chronic pain in adults with ADHD through neuroinflammation treatment [56].

Shifting focus to the post-traumatic inflammatory response, microglia and their associated molecular pathways play a pivotal role [57], especially in scenarios like traumatic brain injury (TBI) [58]. Research indicates that TBI coincides with heightened neuroinflammatory responses, as observed in mice with tibial fractures [59]. These responses are concurrent with disruptions in the blood-brain barrier and behavioral deficits. The increase in interleukin-1β (IL-1β) levels in the brain is associated with these changes, influencing astrocytes, microglia, and neurons while governing neuroinflammatory processes [57, 60]. This series of findings underscores the intricate interplay among fractures, NDDs, inflammation, and pain.

Vitamin D is crucial for both musculoskeletal well-being and brain function. It is indispensable for maintaining healthy bones, but its deficiency can elevate the risk of fractures [61, 62]. In terms of brain function, vitamin D functions as a neurosteroid hormone, influencing neurodevelopment processes such as nerve cell proliferation, neurotransmission, oxidative stress regulation, and immune function—all of which are integral to the central nervous system [63, 64].

Vitamin D deficiency has been linked to various NDDs and psychiatric disorders, including ADHD, ASD, and schizophrenia [65–67]. Individuals with ADHD and ASD frequently exhibit lower levels of 25-hydroxyvitamin D (25(OH)D) in their serum compared to their healthy counterparts [68, 69]. Moreover, an inverse relationship exists between vitamin D and its receptor levels (VDR) and the severity of ASD and ADHD [70, 71]. This suggests that vitamin D may play a significant role not only in the development of ASD but also in ADHD and fractures.

In the context of ASD, children typically engage in less physical activity, have a lower BMI, and may not consume adequate amounts of calcium and calories [72, 73]. These factors collectively increase the risk of low bone density in individuals with ASD [15, 74]. For ADHD, various factors contribute to injuries and fractures in affected children. Some of these incidents can be attributed to the unique characteristics of ADHD [75]. Impulsivity is a hallmark symptom of ADHD, characterized by actions taken without prior thought, insufficient judgment, and a tendency to seek immediate gratification [10, 75]. Research suggests that children with ADHD, despite being capable of recognizing dangers, tend to underestimate the severity of consequences arising from their behaviors [76]. This inherent impulsivity may render children with ADHD more susceptible to injuries and fractures, even in situations comparable to their peers without ADHD [75]. Similarly, low intelligence in the early years has been shown to increase the risk of unintentional injury [77].

However, it is crucial to acknowledge certain limitations in our study. Firstly, we did not analyze the causal relationship between ADHD, ASD, and fractures based on variables such as sex, age, BMI, and other relevant factors. Nevertheless, existing research suggests that these factors could potentially influence causation. Secondly, our study exclusively relied on genetic data from individuals of European ancestry, and we did not validate our findings in non-European populations. As a result, the generalizability of our conclusions to non-European populations remains uncertain.

While our study has several important strengths, providing insights into healthcare management strategies for children with ADHD, it is essential to recognize its limitations. Our findings emphasize the importance of prioritizing the bone health of children with ADHD by monitoring their BMD and implementing appropriate vitamin D supplementation strategies. Furthermore, our results may prompt a shift in the approach to fractures in children. ADHD symptoms are often overlooked in the context of fracture management, and our study aims to address this gap. One key strength of our research lies in the application of the MR method. This approach effectively addresses the potential influence of external social factors on the relationship between exposure variables and outcomes, thereby enhancing the robustness and reliability of our findings.

### Electronic supplementary material

Below is the link to the electronic supplementary material.

**Supplementary Material 1:** Supplementary Material Supplementary Figure 1, 4, 7, 10 and 10-22 showed the leave one out plot of NDDs on bone fractures and osteoporosis; Figure 2, 5, 8 and 22 showed the funnel plot of NDDs on bone fractures and osteoporosis; Figure 3, 6, 9 and 23 showed the scatter plot of NDDs on bone fractures and osteoporosis. Supplementary Figure 24-26 showed the leave one out plot bone fractures and osteoporosis on NDDs; Supplementary Figure 27-29 showed the funnel plot bone fractures and osteoporosis on NDDs; Supplementary Figure 30-32 showed the scatter plot bone fractures and osteoporosis on NDDs

**Supplementary Material 2:** Table S1, Summary data for exposure and outcome; Table S2, The IVs for our study; Table S3, The results for causal effect; Table S4, Results of heterogeneity test; Table S5, Results of sensitivity analysis

## Data Availability

Summary data on ADHD, ASD, TS, Fractures and Osteoporosis is available on supplementary material.
