# Supplementary material for: Complex interplay of neurodevelopmental disorders (NDDs), fractures, and osteoporosis: a mendelian randomization study
Source: BMC Psychiatry. 2024 Mar 27;24:232. doi: 10.1186/s12888-024-05693-4 (PMC10967110; doi:10.1186/s12888-024-05693-4)
Supplement: Supplementary file 1 — Supplementary Material 1: Supplementary Material Supplementary Figure 1, 4, 7, 10 and 10-22 showed the leave one out plot of NDDs on bone fractures and osteoporosis; Figure 2, 5, 8 and 22 showed the funnel plot of NDDs on bone fractures and osteoporosis; Figure 3, 6, 9 and 23 showed the scatter plot of NDDs on bone fractures and osteoporosis. Supplementary Figure 24-26 showed the leave one out plot bone fractures and osteoporosis on NDDs; Supplementary Figure 27-29 showed the funnel plot bone fractures and osteoporosis on NDDs; Supplementary Figure 30-32 showed the scatter plot bone fractures and osteoporosis on NDDs [file 12888_2024_5693_MOESM1_ESM.pdf]

Catalogue for supplementary figure:

The leave one out plot of the impact of ADHD on bone fractures and osteoporosis……………………….1
The funnel plot of the impact of ADHD on bone fractures and osteoporosis……………………………2
The scatter plot of the impact of ADHD on bone fractures and osteoporosis……………………………3
The leave one out plot of the impact of ASD on bone fractures and osteoporosis ………………………4
The funnel plot of the impact of ASD on bone fractures and osteoporosis………………………………5
The scatter plot of the impact of ASD on bone fractures and osteoporosis………………………………6
The leave one out plot of the impact of TS on bone fractures and osteoporosis…………………………7
The funnel plot of the impact of TS on bone fractures and osteoporosis………………………………...8
The scatter plot of the impact of TS on bone fractures and osteoporosis…………………………………9
The leave one out plot of the impact of Intelligence on bone fractures and osteoporosis…………...10-21
The funnel plot of the impact of Intelligence on bone fractures and osteoporosis……………………….22
The scatter plot of the impact of Intelligence on bone fractures and osteoporosis………………………23
The leave one out plot of the impact of bone fractures and osteoporosis on NDDs…………………24-26
The funnel plot of the impact of bone fractures and osteoporosis on NDDs……………………...…27-29
The scatter plot of the impact of bone fractures and osteoporosis on NDDs………………………...30-32

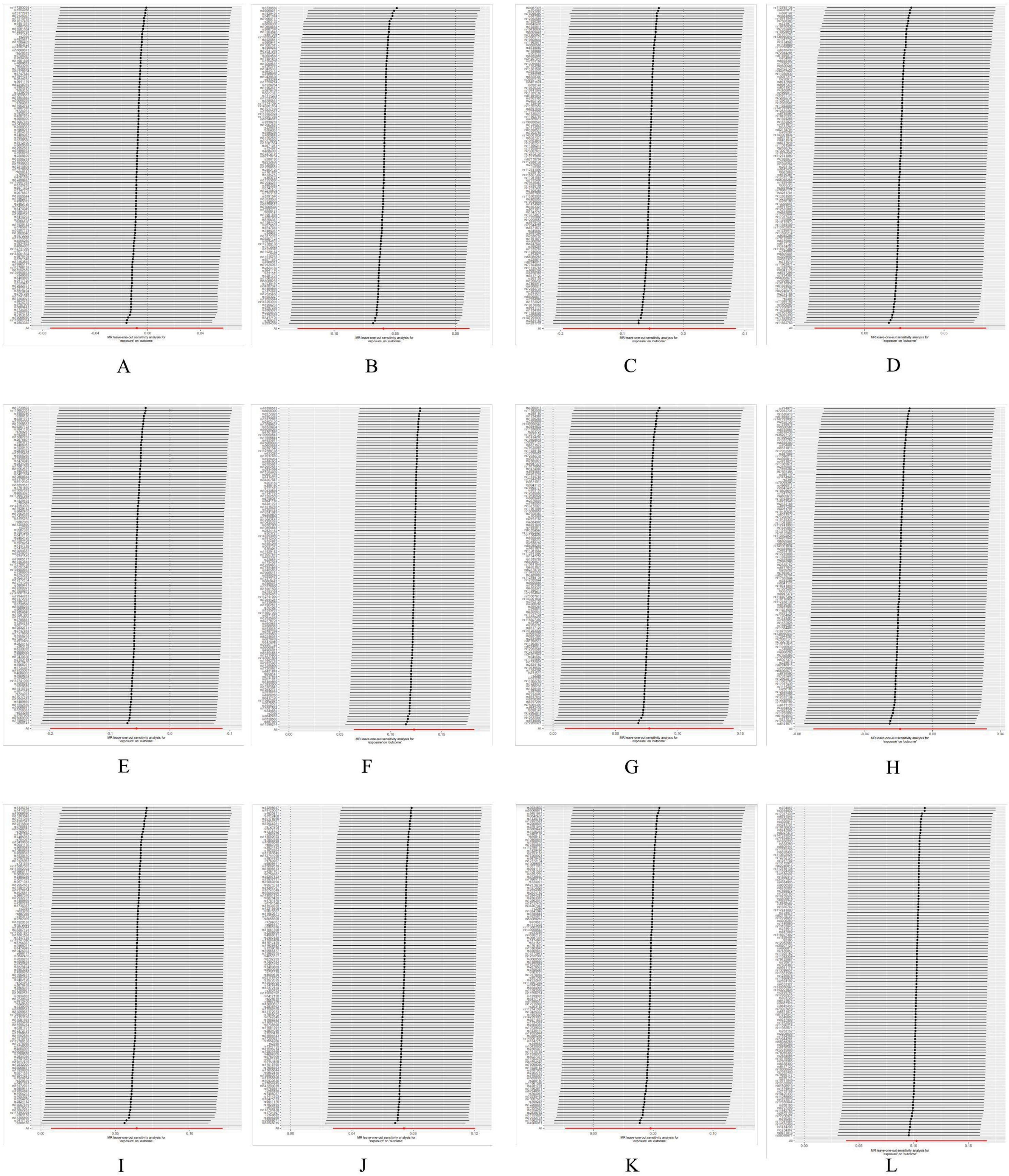

THE LEAVE ONE OUT PLOT OF THE IMPACT OF TS ON BONE FRACTURES AND OSTEOPOROSIS
E.G(EXPOSURE-OUTCOME)
A: (ADHD-FRACTURE OF FEMUR); B: (ADHD-OSTEOPOROSIS);
C: (ADHD-OSTEOPOROSIS WITH PATHOLOGICAL FRACTURE (FG));
D: (ADHD-FRACTURE OF SHOULDER AND UPPER ARM);E : (ADHD-FRACTURE OF NECK);
F: (ADHD-FRACTURE OF RIB(S), STERNUM AND THORACIC SPINE);
G: (ADHD-FRACTURE OF SKULL AND FACIAL BONES); H: (ADHD-FRACTURE OF FOREARM);
I: (ADHD-FRACTURE AT WRIST AND HAND LEVEL); G: (ADHD-FRACTURE OF LOWER LEG, INCLUDING ANKLE);
K: (ADHD-FRACTURE OF LUMBAR SPINE AND PELVIS); L: (ADHD-FRACTURE OF FOOT, EXCEPT ANKLE)

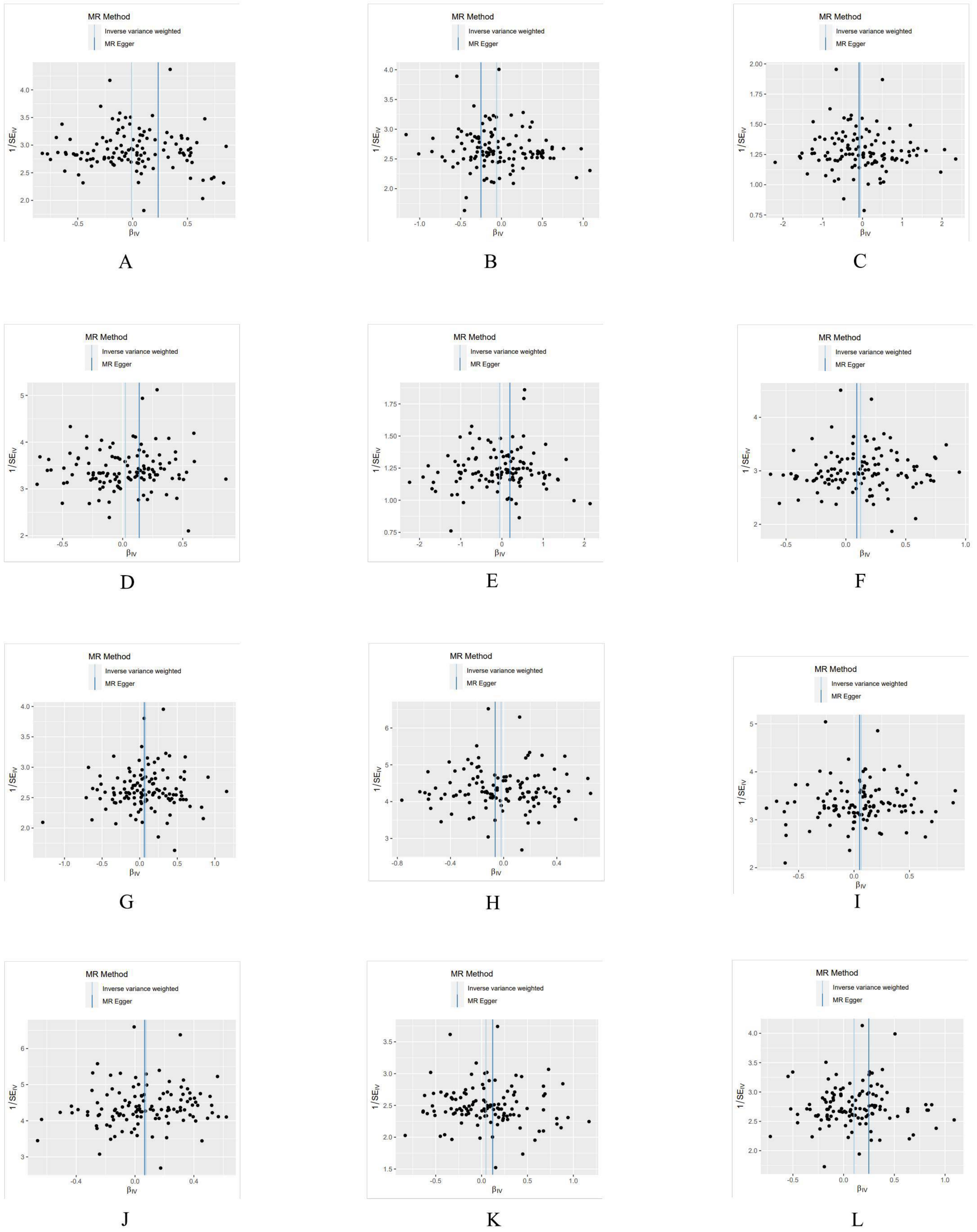

THE FUNNEL PLOT OF THE IMPACT OF ADHD ON BONE FRACTURES AND OSTEOPOROSIS
E.G(EXPOSURE-OUTCOME)
A: (ADHD-FRACTURE OF FEMUR); B: (ADHD-OSTEOPOROSIS);
C: (ADHD-OSTEOPOROSIS WITH PATHOLOGICAL FRACTURE (FG));
D: (ADHD-FRACTURE OF SHOULDER AND UPPER ARM);E : (ADHD-FRACTURE OF NECK);
F: (ADHD-FRACTURE OF RIB(S), STERNUM AND THORACIC SPINE);
G: (ADHD-FRACTURE OF SKULL AND FACIAL BONES); H: (ADHD-FRACTURE OF FOREARM);
I: (ADHD-FRACTURE AT WRIST AND HAND LEVEL); G: (ADHD-FRACTURE OF LOWER LEG, INCLUDING ANKLE);
K: (ADHD-FRACTURE OF LUMBAR SPINE AND PELVIS); L: (ADHD-FRACTURE OF FOOT, EXCEPT ANKLE)

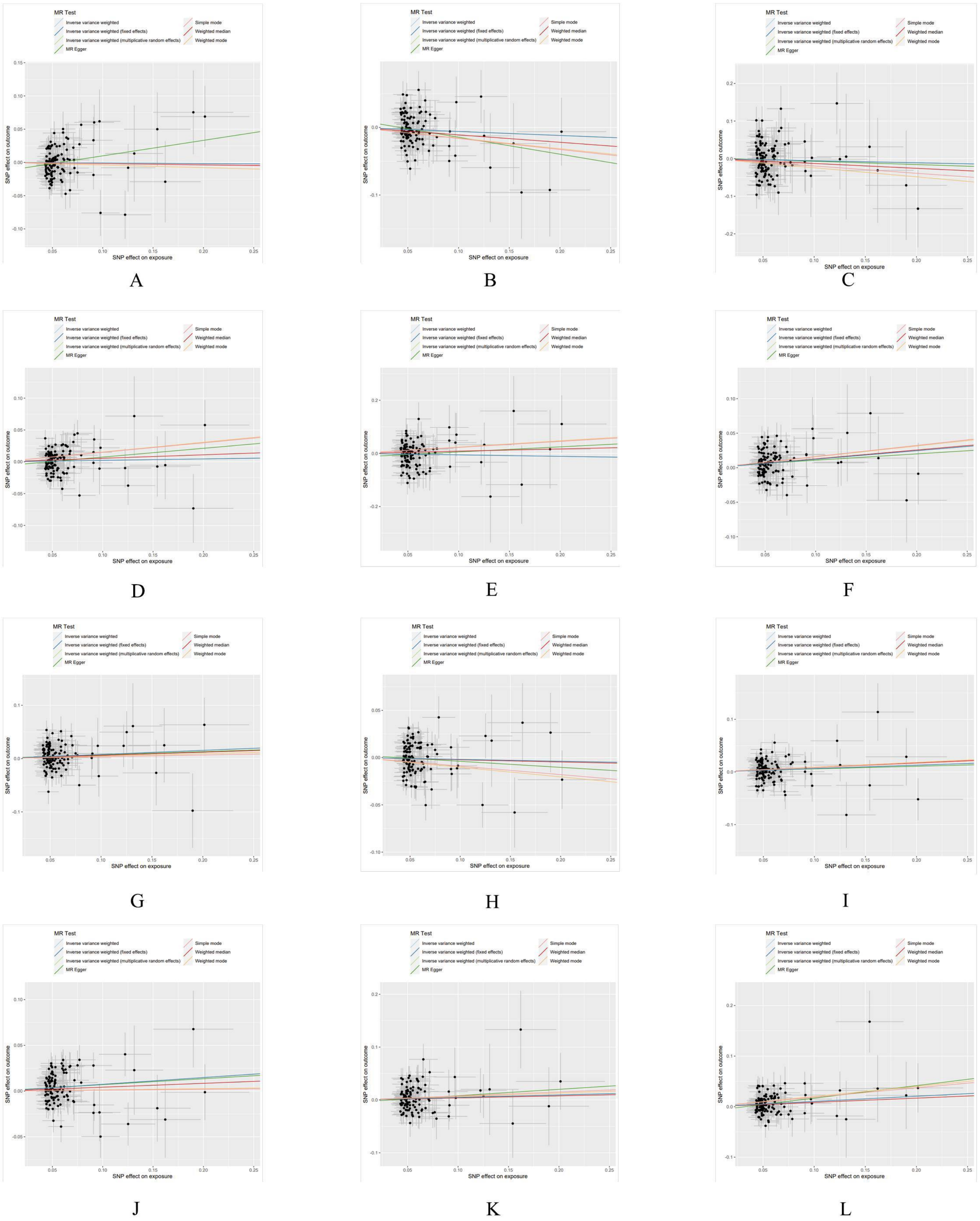

THE FUNNEL PLOT OF THE IMPACT OF ADHD ON BONE FRACTURES AND OSTEOPOROSIS
E.G(EXPOSURE-OUTCOME)
A: (ADHD-FRACTURE OF FEMUR); B: (ADHD-OSTEOPOROSIS);
C: (ADHD-OSTEOPOROSIS WITH PATHOLOGICAL FRACTURE (FG));
D: (ADHD-FRACTURE OF SHOULDER AND UPPER ARM);E : (ADHD-FRACTURE OF NECK);
F: (ADHD-FRACTURE OF RIB(S), STERNUM AND THORACIC SPINE);
G: (ADHD-FRACTURE OF SKULL AND FACIAL BONES); H: (ADHD-FRACTURE OF FOREARM);
I: (ADHD-FRACTURE AT WRIST AND HAND LEVEL); G: (ADHD-FRACTURE OF LOWER LEG, INCLUDING ANKLE);
K: (ADHD-FRACTURE OF LUMBAR SPINE AND PELVIS); L: (ADHD-FRACTURE OF FOOT, EXCEPT ANKLE)

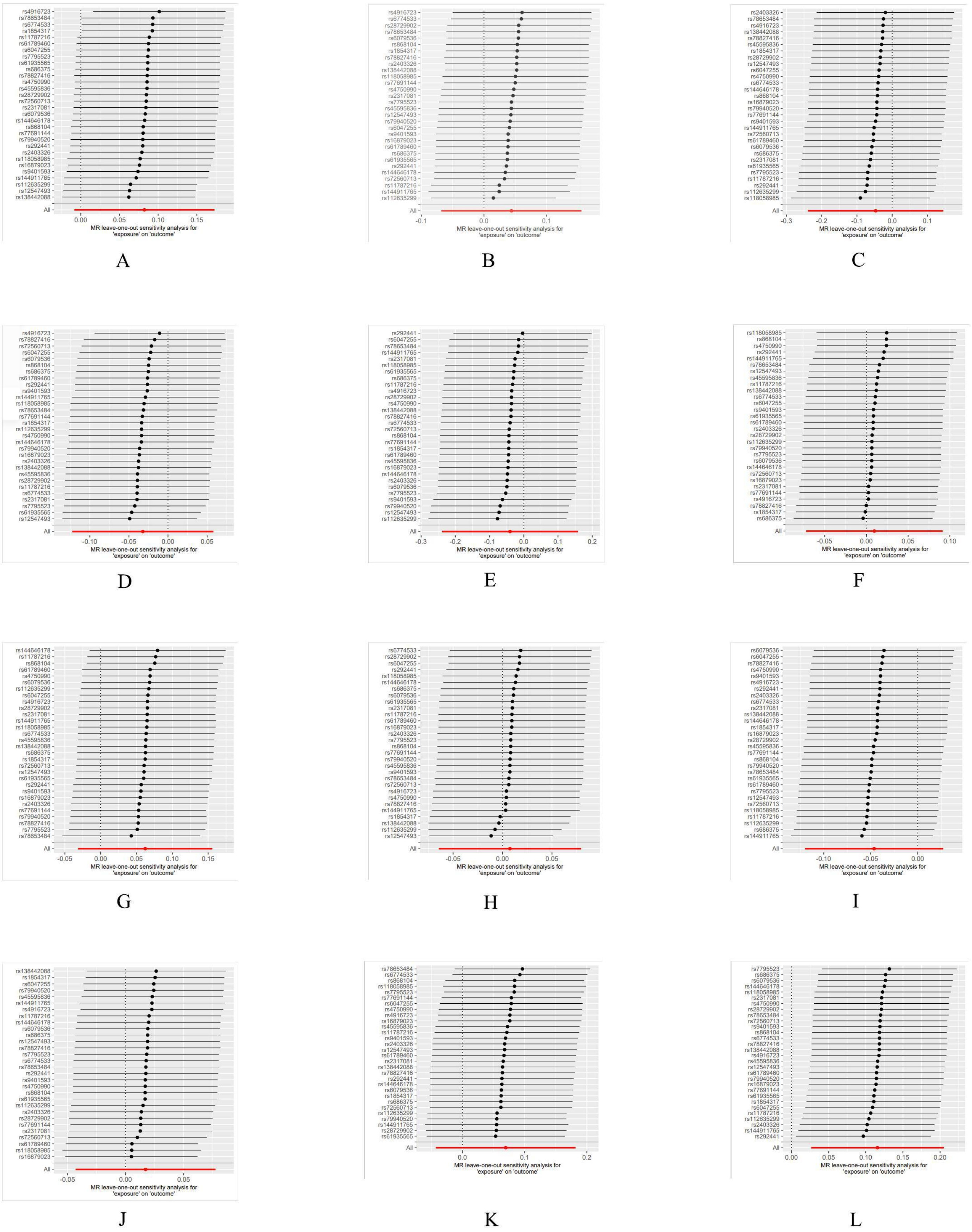

THE LEAVE ONE OUT PLOT OF THE IMPACT OF TS ON BONE FRACTURES AND OSTEOPOROSIS E.G(EXPOSURE-OUTCOME)

A: (ASD-FRACTURE OF FEMUR); B: (ASD-OSTEOPOROSIS);

C: (ASD-OSTEOPOROSIS WITH PATHOLOGICAL FRACTURE (FG));

D: (ASD-FRACTURE OF SHOULDER AND UPPER ARM);E : (ASD-FRACTURE OF NECK);

F: (ASD-FRACTURE OF RIB(S), STERNUM AND THORACIC SPINE);

G: (ASD-FRACTURE OF SKULL AND FACIAL BONES); H: (ASD-FRACTURE OF FOREARM);

I: (ASD-FRACTURE AT WRIST AND HAND LEVEL); G: (ASD-FRACTURE OF LOWER LEG, INCLUDING ANKLE);

K: (ASD-FRACTURE OF LUMBAR SPINE AND PELVIS); L: (ASD-FRACTURE OF FOOT, EXCEPT ANKLE)

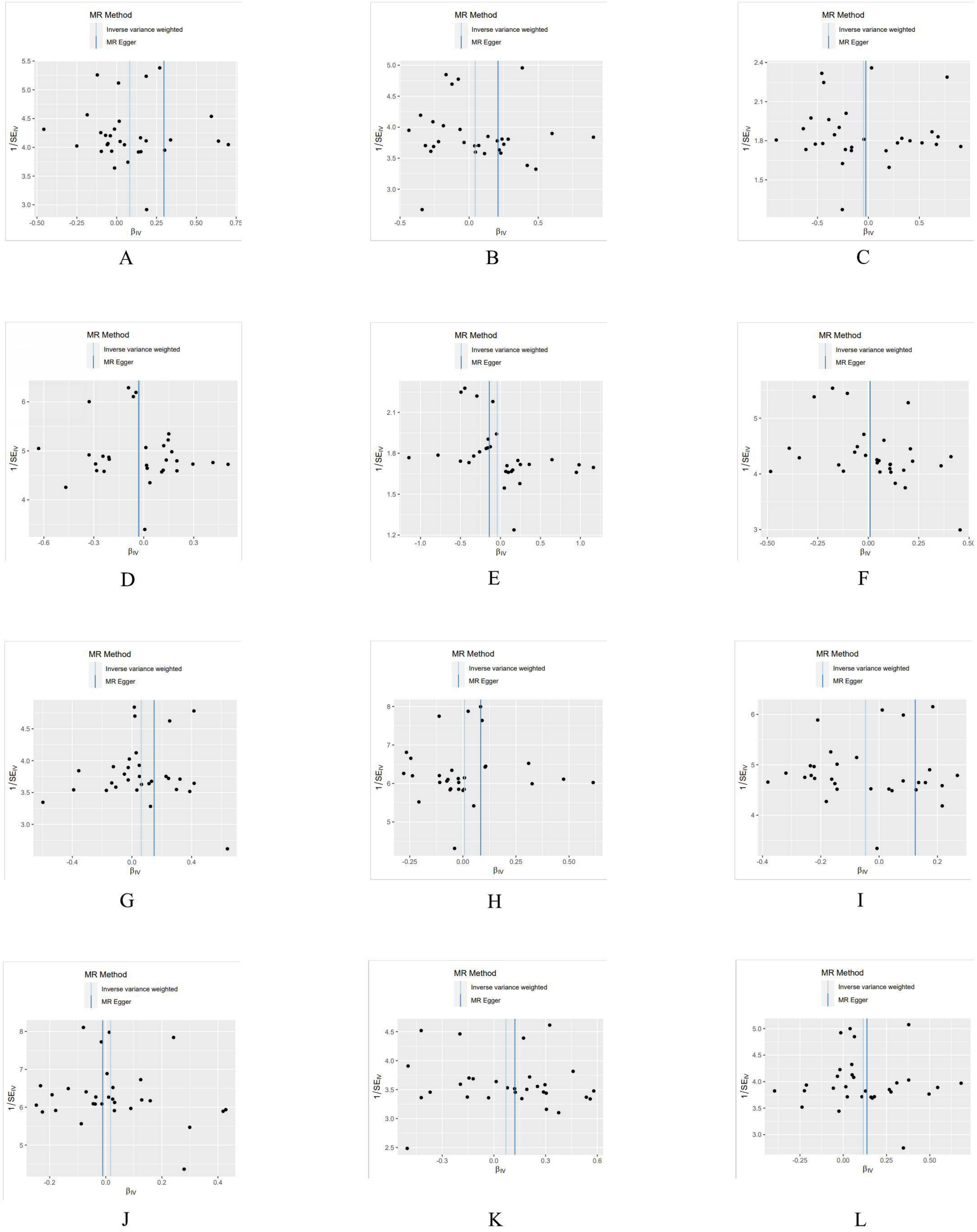

THE FUNNEL PLOT OF THE IMPACT OF ASD ON BONE FRACTURES AND OSTEOPOROSIS
E.G(EXPOSURE-OUTCOME)
A: (ASD-FRACTURE OF FEMUR); B: (ASD-OSTEOPOROSIS);
C: (ASD-OSTEOPOROSIS WITH PATHOLOGICAL FRACTURE (FG));
D: (ASD-FRACTURE OF SHOULDER AND UPPER ARM);E : (ASD-FRACTURE OF NECK);
F: (ASD-FRACTURE OF RIB(S), STERNUM AND THORACIC SPINE);
G: (ASD-FRACTURE OF SKULL AND FACIAL BONES); H: (ASD-FRACTURE OF FOREARM);
I: (ASD-FRACTURE AT WRIST AND HAND LEVEL); G: (ASD-FRACTURE OF LOWER LEG, INCLUDING ANKLE);
K: (ASD-FRACTURE OF LUMBAR SPINE AND PELVIS); L: (ASD-FRACTURE OF FOOT, EXCEPT ANKLE)

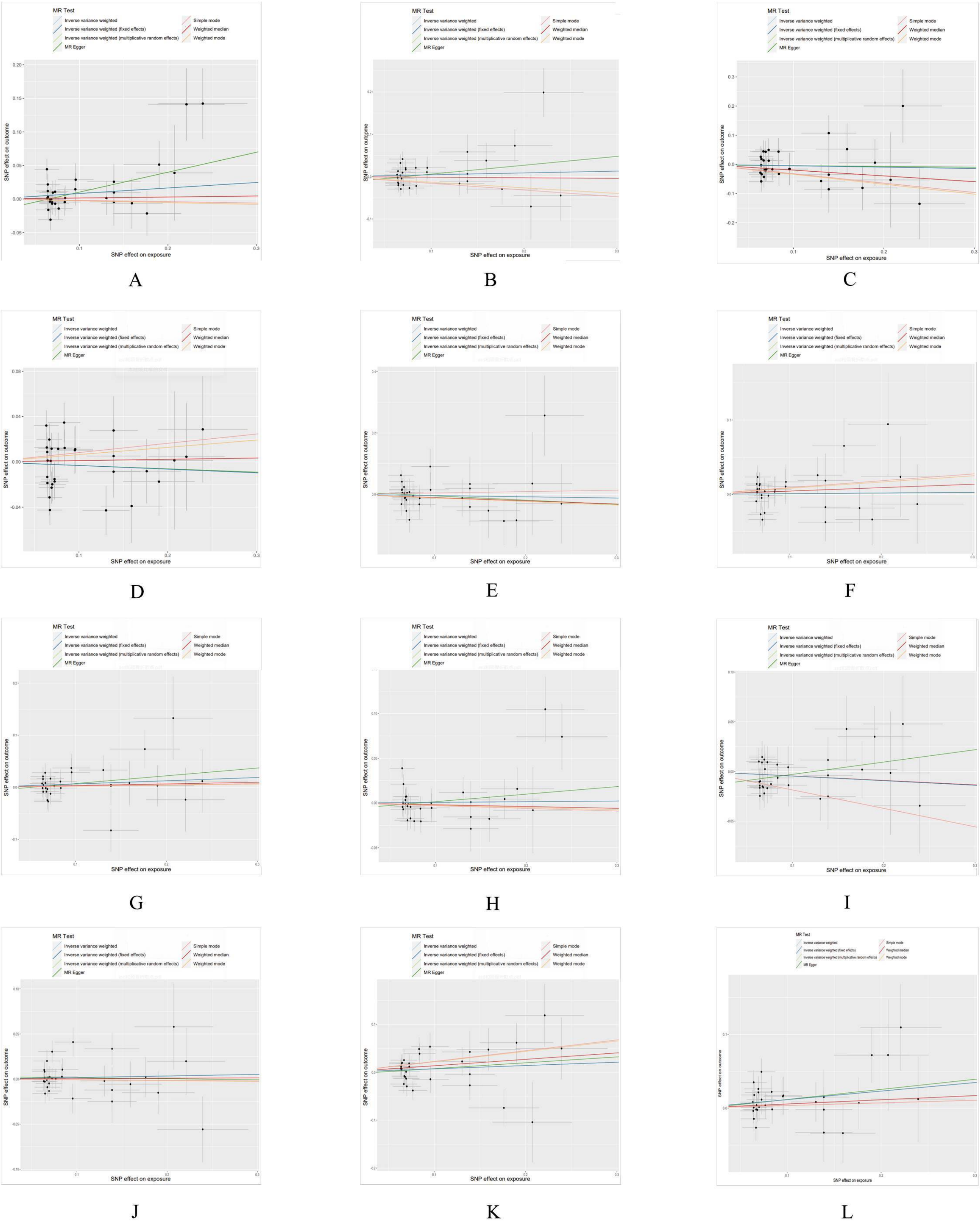

THE FUNNEL PLOT OF THE IMPACT OF ASD ON BONE FRACTURES AND OSTEOPOROSIS

E.G(EXPOSURE-OUTCOME)
A: (ASD-FRACTURE OF FEMUR); B: (ASD-OSTEOPOROSIS);
C: (ASD-OSTEOPOROSIS WITH PATHOLOGICAL FRACTURE (FG));
D: (ASD-FRACTURE OF SHOULDER AND UPPER ARM);E : (ASD-FRACTURE OF NECK);
F: (ASD-FRACTURE OF RIB(S), STERNUM AND THORACIC SPINE);
G: (ASD-FRACTURE OF SKULL AND FACIAL BONES); H: (ASD-FRACTURE OF FOREARM);
I : (ASD-FRACTURE AT WRIST AND HAND LEVEL); G: (ASD-FRACTURE OF LOWER LEG, INCLUDING ANKLE);
K: (ASD-FRACTURE OF LUMBAR SPINE AND PELVIS); L: (ASD-FRACTURE OF FOOT, EXCEPT ANKLE)

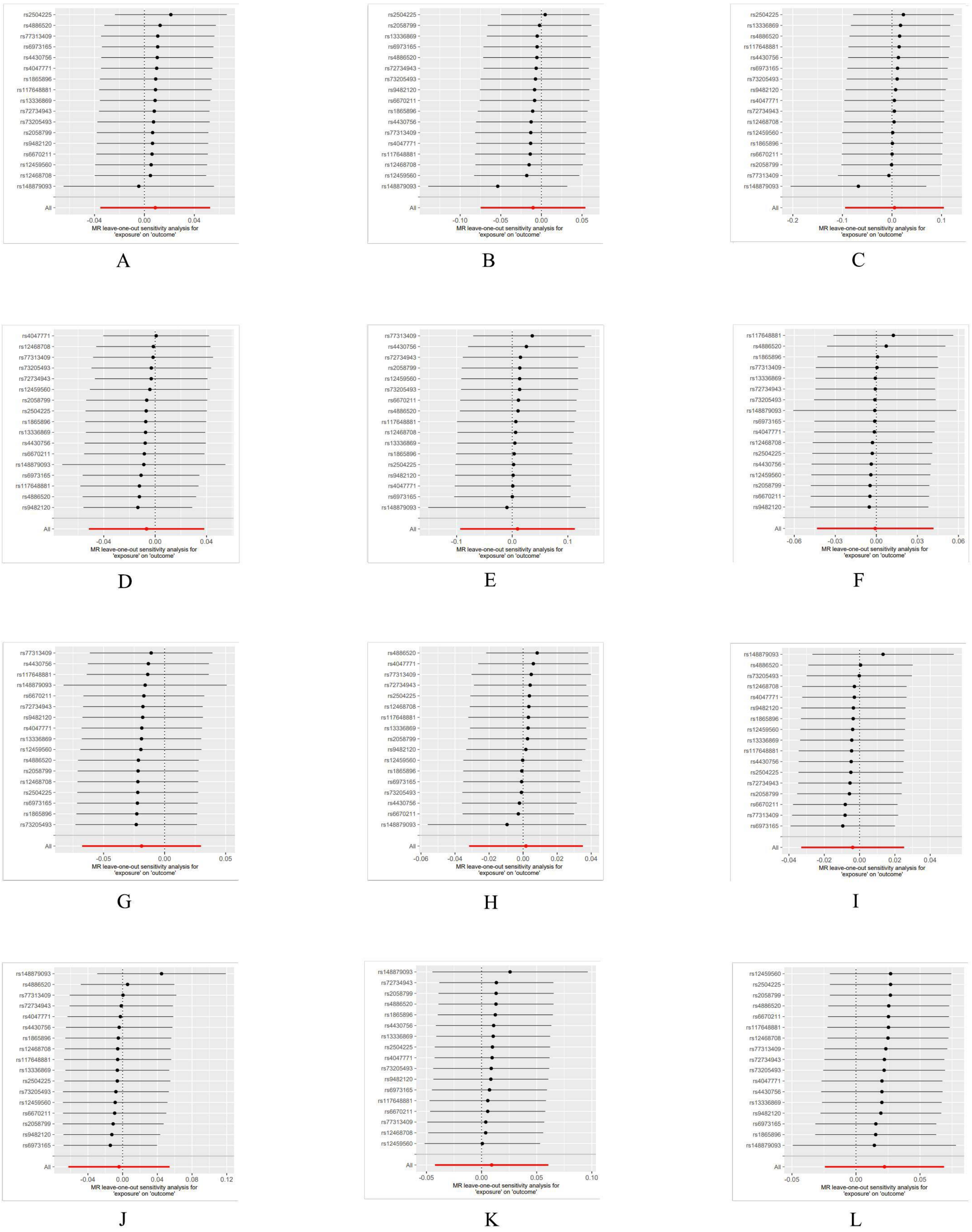

THE LEAVE ONE OUT PLOT OF THE IMPACT OF TS ON BONE FRACTURES AND OSTEOPOROSIS E.G(EXPOSURE-OUTCOME)

A: (TS-FRACTURE OF FEMUR); B: (TS-OSTEOPOROSIS);

C: (TS-OSTEOPOROSIS WITH PATHOLOGICAL FRACTURE (FG));

D: (TS-FRACTURE OF SHOULDER AND UPPER ARM);E : (TS-FRACTURE OF NECK);

F: (TS-FRACTURE OF RIB(S), STERNUM AND THORACIC SPINE);

G: (TS-FRACTURE OF SKULL AND FACIAL BONES); H: (TS-FRACTURE OF FOREARM);

I: (TS-FRACTURE AT WRIST AND HAND LEVEL); G: (TS-FRACTURE OF LOWER LEG, INCLUDING ANKLE);

K: (TS-FRACTURE OF LUMBAR SPINE AND PELVIS); L: (TS-FRACTURE OF FOOT, EXCEPT ANKLE)

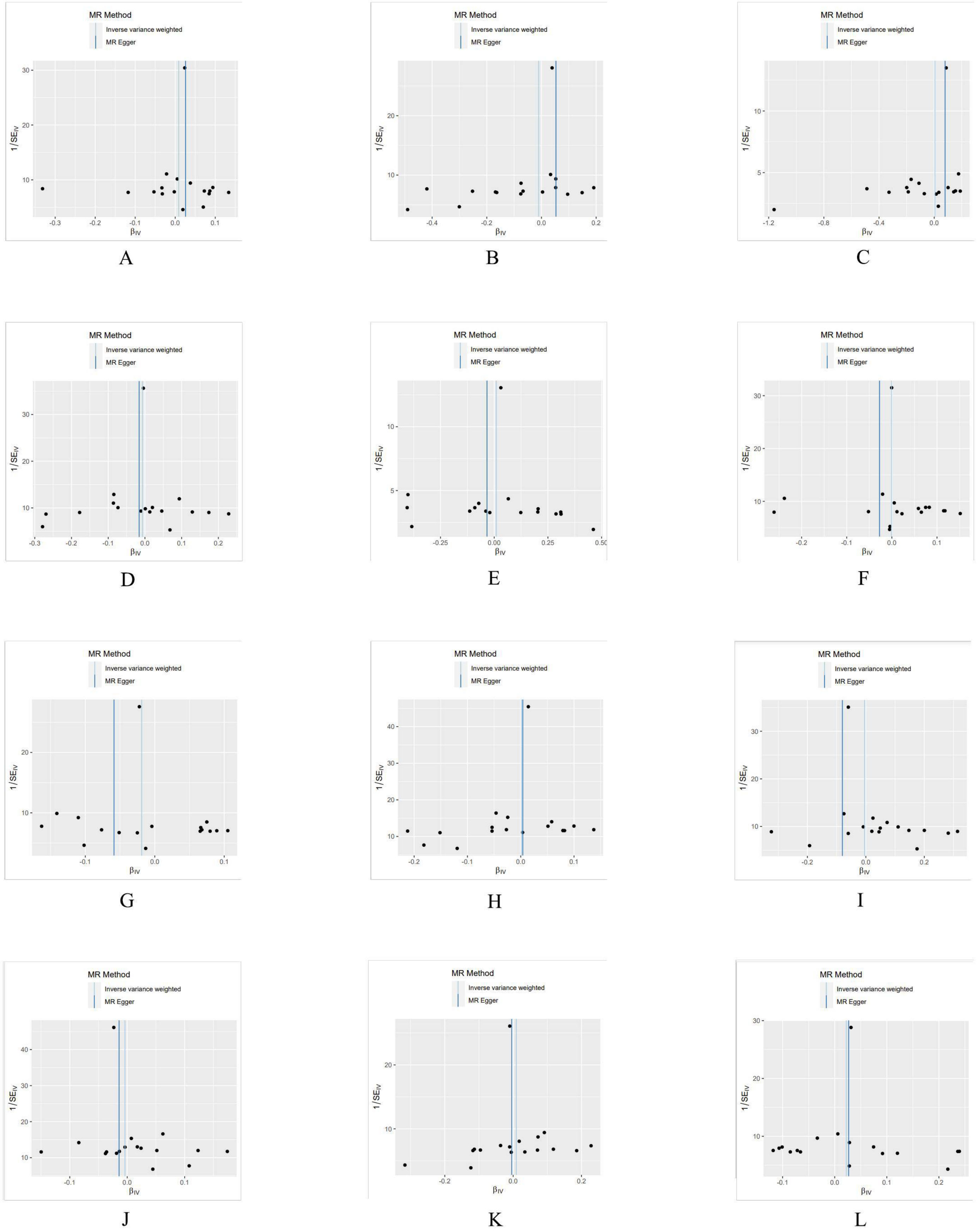

THE FUNNEL PLOT OF THE IMPACT OF TS ON BONE FRACTURES AND OSTEOPOROSIS
E.G(EXPOSURE-OUTCOME)
A: (TS-FRACTURE OF FEMUR); B: (TS-OSTEOPOROSIS);
C: (TS-OSTEOPOROSIS WITH PATHOLOGICAL FRACTURE (FG));
D: (TS-FRACTURE OF SHOULDER AND UPPER ARM);E : (TS-FRACTURE OF NECK);
F: (TS-FRACTURE OF RIB(S), STERNUM AND THORACIC SPINE);
G: (TS-FRACTURE OF SKULL AND FACIAL BONES); H: (TS-FRACTURE OF FOREARM);
I: (TS-FRACTURE AT WRIST AND HAND LEVEL); G: (TS-FRACTURE OF LOWER LEG, INCLUDING ANKLE);
K: (TS-FRACTURE OF LUMBAR SPINE AND PELVIS); L: (TS-FRACTURE OF FOOT, EXCEPT ANKLE)

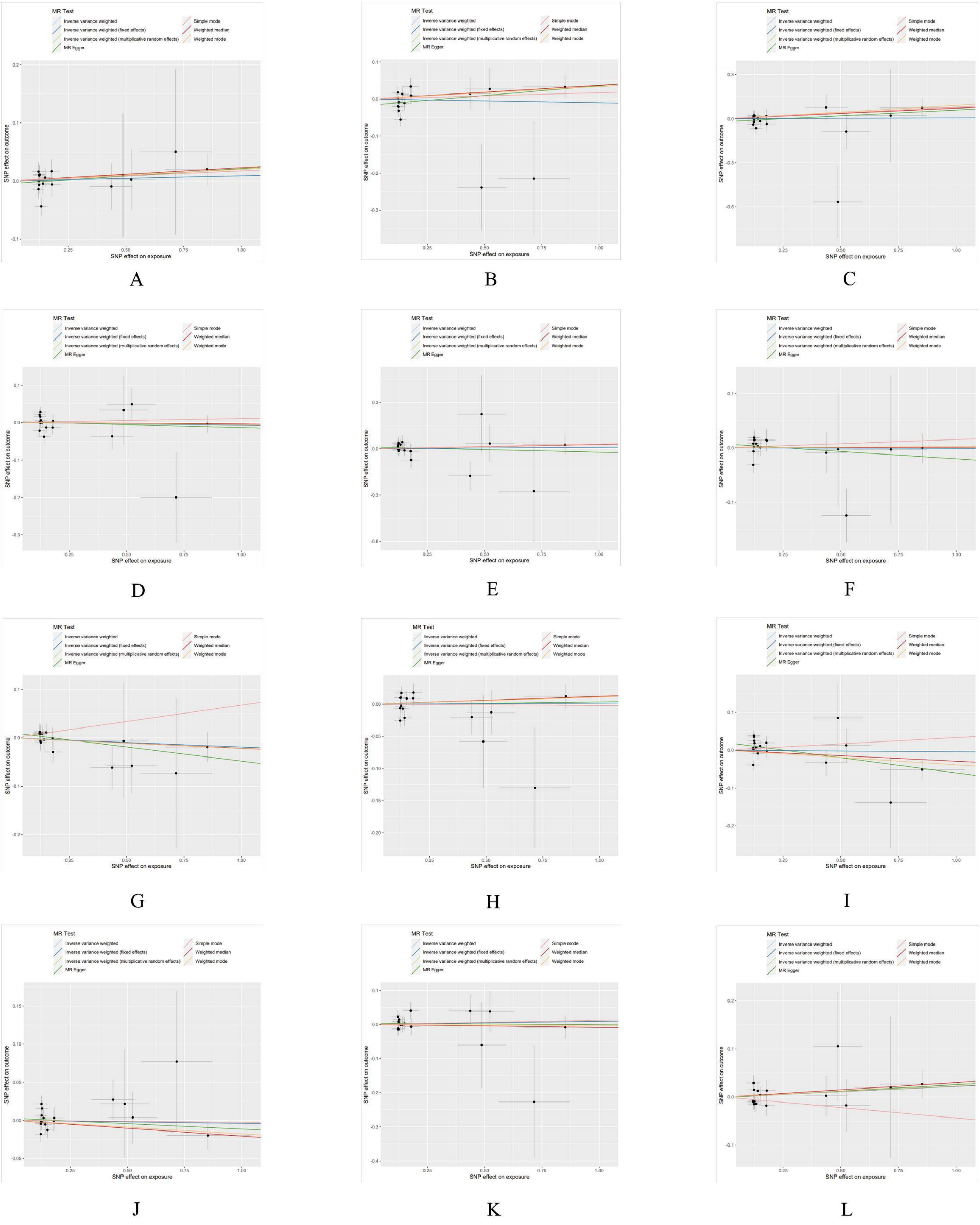

THE FUNNEL PLOT OF THE IMPACT OF BONE FRACTURES AND OSTEOPOROSISON ON THREE PSYCHIATRIC DISORDERS

E.G(EXPOSURE-OUTCOME)

A: (TS-FRACTURE OF FEMUR); B: (TS-OSTEOPOROSIS);

C: (TS-OSTEOPOROSIS WITH PATHOLOGICAL FRACTURE (FG));

D: (TS-FRACTURE OF SHOULDER AND UPPER ARM);E : (TS-FRACTURE OF NECK);

F: (TS-FRACTURE OF RIB(S), STERNUM AND THORACIC SPINE);

G: (TS-FRACTURE OF SKULL AND FACIAL BONES); H: (TS-FRACTURE OF FOREARM);

I : (TS-FRACTURE AT WRIST AND HAND LEVEL); G: (TS-FRACTURE OF LOWER LEG, INCLUDING ANKLE);

K: (TSD-FRACTURE OF LUMBAR SPINE AND PELVIS); L: (TSD-FRACTURE OF FOOT, EXCEPT ANKLE)

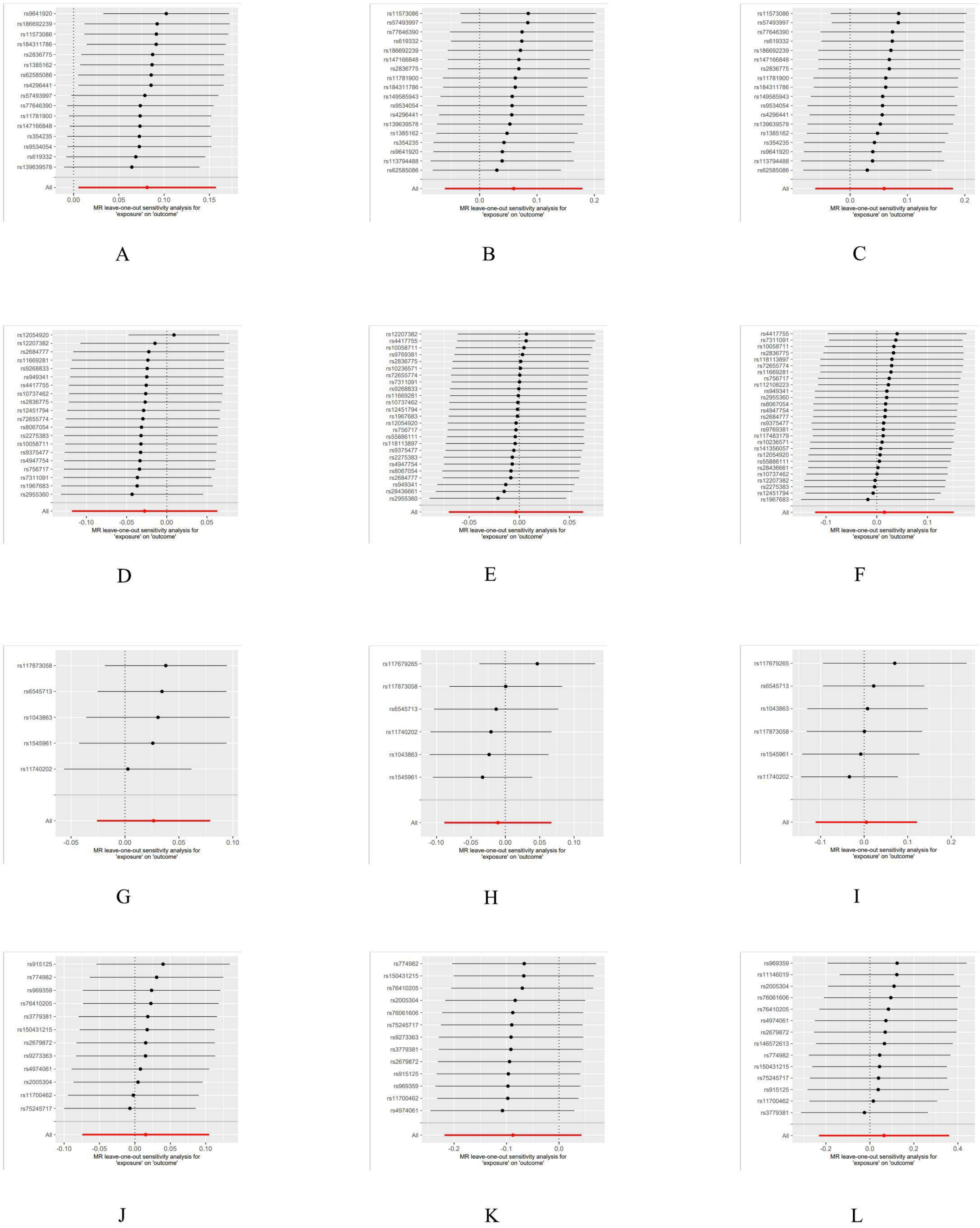

THE LEAVE ONE OUT PLOT OF THE IMPACT OF BONE FRACTURES AND OSTEOPOROSIS ON THREE PSYCHIATRIC DISORDERS

E.G(EXPOSURE-OUTCOME)

A: (FRACTURE OF FEMUR-ADHD); B: (FRACTURE OF FEMUR-ASD);

C: (FRACTURE OF FEMUR-TS); D: (OSTEOPOROSIS-ADHD);E: (OSTEOPOROSIS-ASD);F: (OSTEOPOROSIS-TS);

G: (OSTEOPOROSIS WITH PATHOLOGICAL FRACTURE (FG)-ADHD);

H: (OSTEOPOROSIS WITH PATHOLOGICAL FRACTURE (FG)-ASD);

I: (OSTEOPOROSIS WITH PATHOLOGICAL FRACTURE (FG)-TS);

G: (FRACTURE OF SHOULDER AND UPPER ARM-ADHD); K: (FRACTURE OF SHOULDER AND UPPER ARM-ASD);

L: (FRACTURE OF SHOULDER AND UPPER ARM-TS)

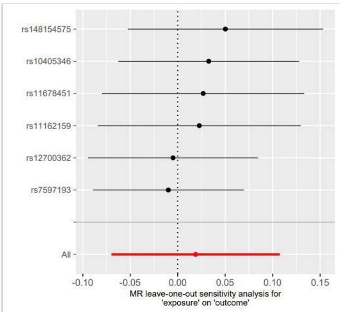

A

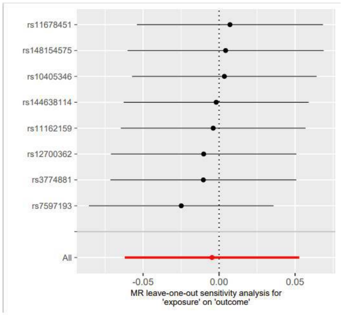

B

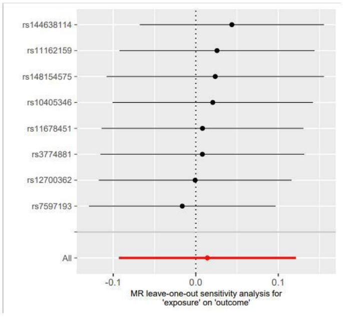

C

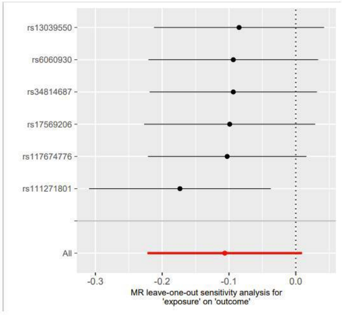

D

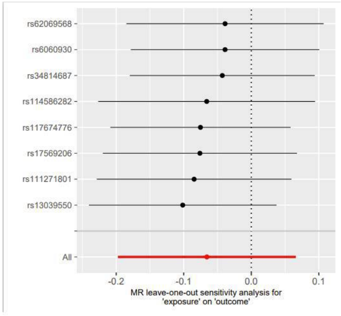

E

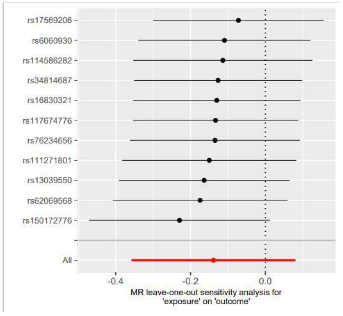

F

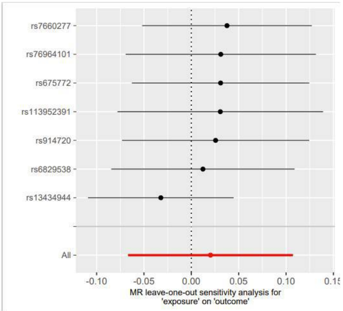

G

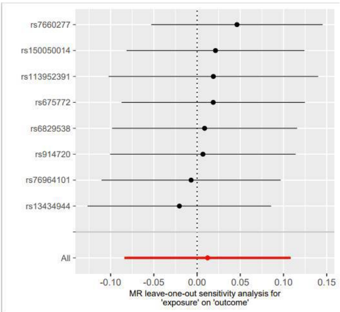

H

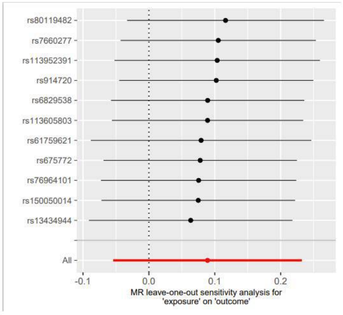

I

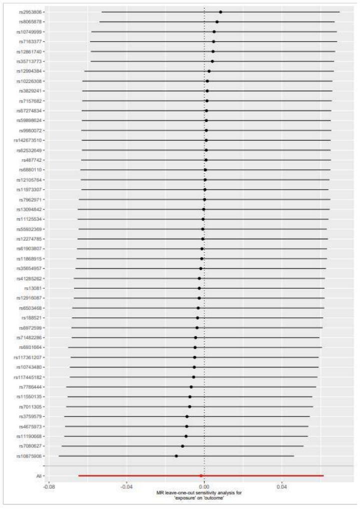

J

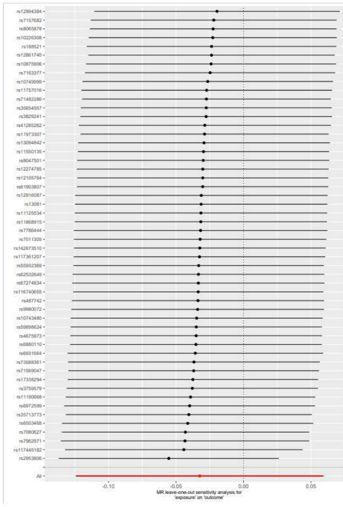

K

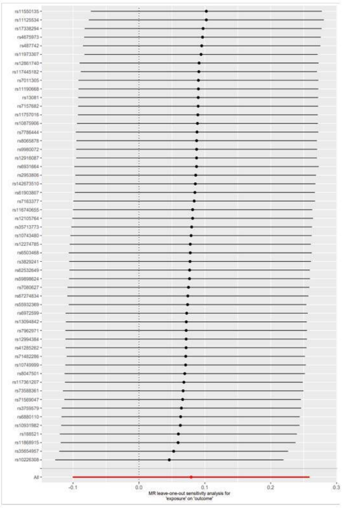

L

THE LEAVE ONE OUT PLOT OF THE IMPACT OF BONE FRACTURES AND OSTEOPOROSIS ON THREE PSYCHIATRIC DISORDERS

E.G(EXPOSURE-OUTCOME)

A: (FRACTURE OF NECK-ADHD); B: (FRACTURE OF NECK-ASD);
C: (FRACTURE OF NECK-TS); D: (FRACTURE OF RIB(S), STERNUM AND THORACIC SPINE-ADHD);
E: (FRACTURE OF RIB(S), STERNUM AND THORACIC SPINE-ASD);
F: (FRACTURE OF RIB(S), STERNUM AND THORACIC SPINE-TS);
G: (FRACTURE OF SKULL AND FACIAL BONES-ADHD); H: (FRACTURE OF SKULL AND FACIAL BONES-ASD);
I : (FRACTURE OF SKULL AND FACIAL BONES-TS); G: (FRACTURE OF FOREARM-ADHD);
K: (FRACTURE OF FOREARM-ASD); L: (FRACTURE OF FOREARM-TS)

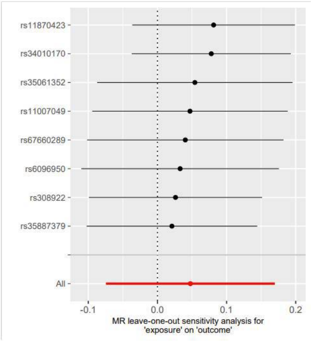

A

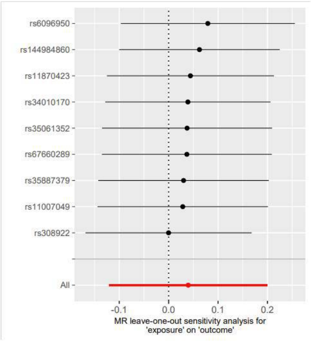

B

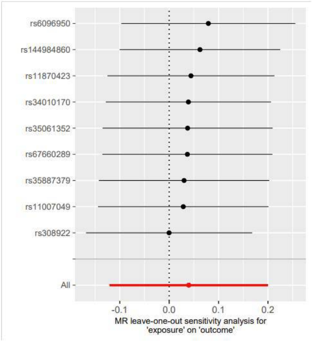

C

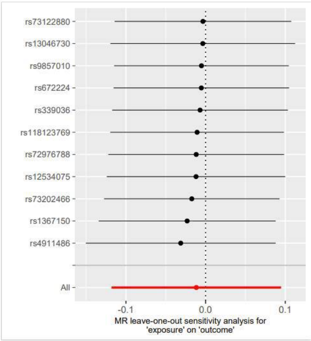

D

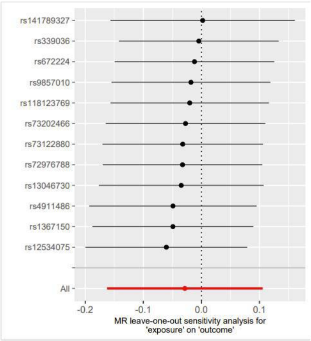

E

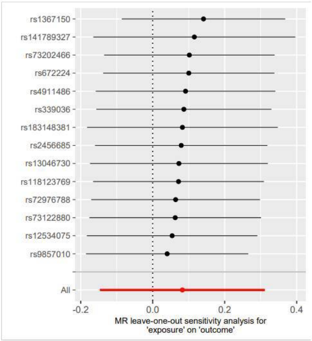

F

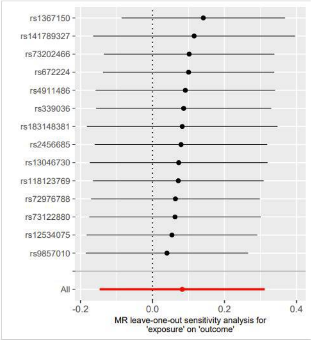

G

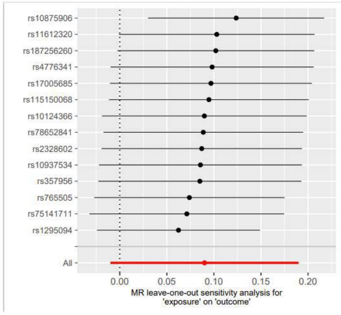

H

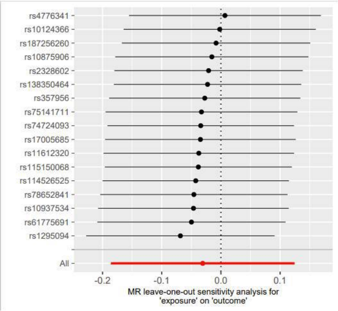

I

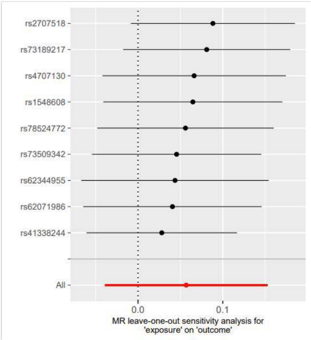

J

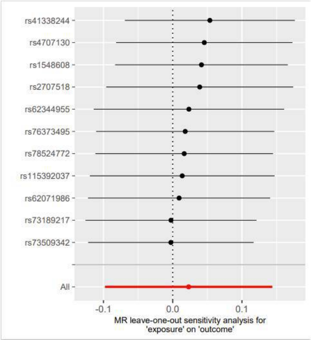

K

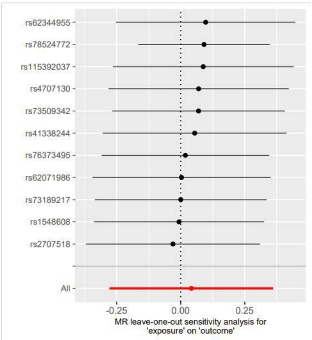

L

THE LEAVE ONE OUT PLOT OF THE IMPACT OF BONE FRACTURES AND OSTEOPOROSIS ON THREE PSYCHIATRIC DISORDERS

E.G(EXPOSURE-OUTCOME)

A: (FRACTURE AT WRIST AND HAND LEVEL-ADHD); B: (FRACTURE AT WRIST AND HAND LEVEL-ASD);
C: (FRACTURE AT WRIST AND HAND LEVEL-TS); D: (FRACTURE OF LOWER LEG, INCLUDING ANKLE-ADHD);
E: (FRACTURE OF LOWER LEG, INCLUDING ANKLE-ASD);
F: (FRACTURE OF LOWER LEG, INCLUDING ANKLE-TS);
G: (FRACTURE OF LUMBAR SPINE AND PELVIS-ADHD); H: (FRACTURE OF LUMBAR SPINE AND PELVIS-ASD);
I : (FRACTURE OF LUMBAR SPINE AND PELVIS-TS); G: (FRACTURE OF FOOT, EXCEPT ANKLE-ADHD);
K: (FRACTURE OF FOOT, EXCEPT ANKLE-ASD); L: (FFRACTURE OF FOOT, EXCEPT ANKLE-TS)

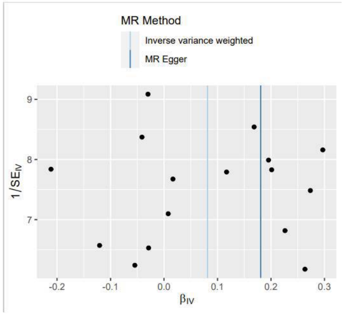

A

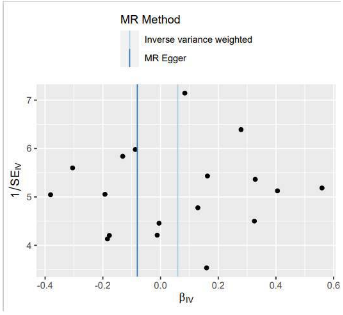

B

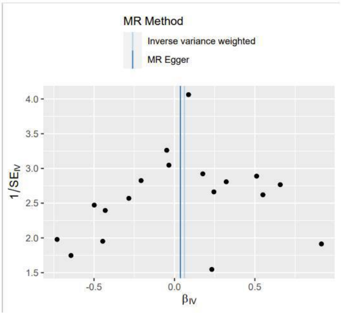

C

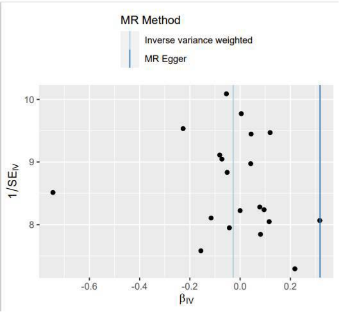

D

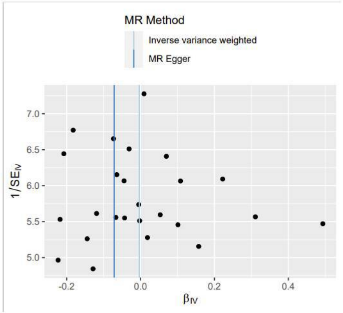

E

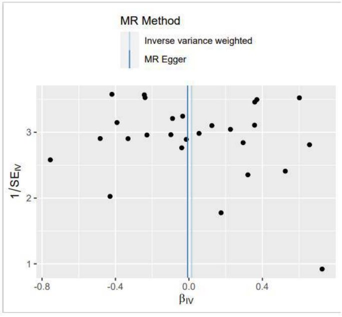

F

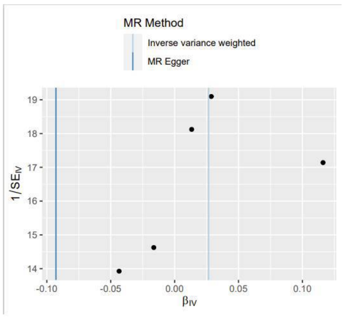

G

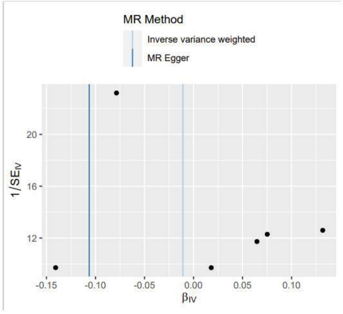

H

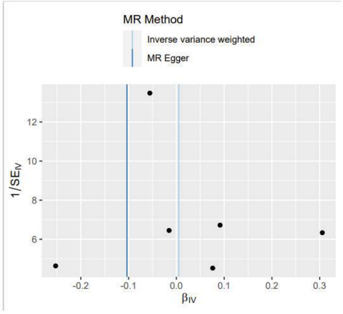

I

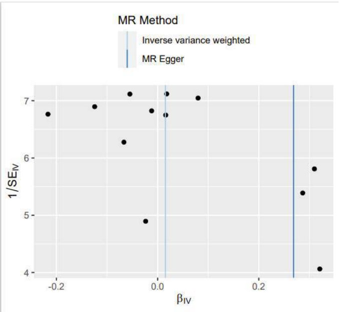

J

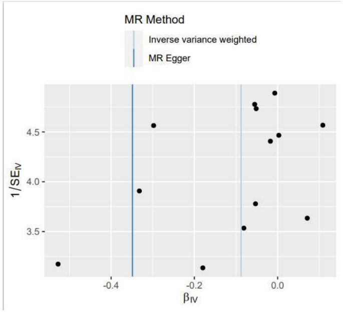

K

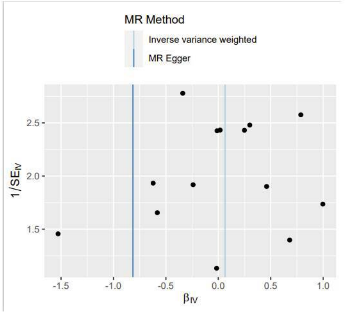

L

THE FUNNEL PLOT OF THE IMPACT OF BONE FRACTURES AND OSTEOPOROSIS ON THREE PSYCHIATRIC DISORDERS

E.G(EXPOSURE-OUTCOME)

A: (FRACTURE OF FEMUR-ADHD); B: (FRACTURE OF FEMUR-ASD);
C: (FRACTURE OF FEMUR-TS); D: (OSTEOPOROSIS-ADHD);E: (OSTEOPOROSIS-ASD);F: (OSTEOPOROSIS-TS);
G: (OSTEOPOROSIS WITH PATHOLOGICAL FRACTURE (FG)-ADHD);
H: (OSTEOPOROSIS WITH PATHOLOGICAL FRACTURE (FG)-ASD);
I: (OSTEOPOROSIS WITH PATHOLOGICAL FRACTURE (FG)-TS);
G: (FRACTURE OF SHOULDER AND UPPER ARM-ADHD); K: (FRACTURE OF SHOULDER AND UPPER ARM-ASD);
L: (FRACTURE OF SHOULDER AND UPPER ARM-TS)

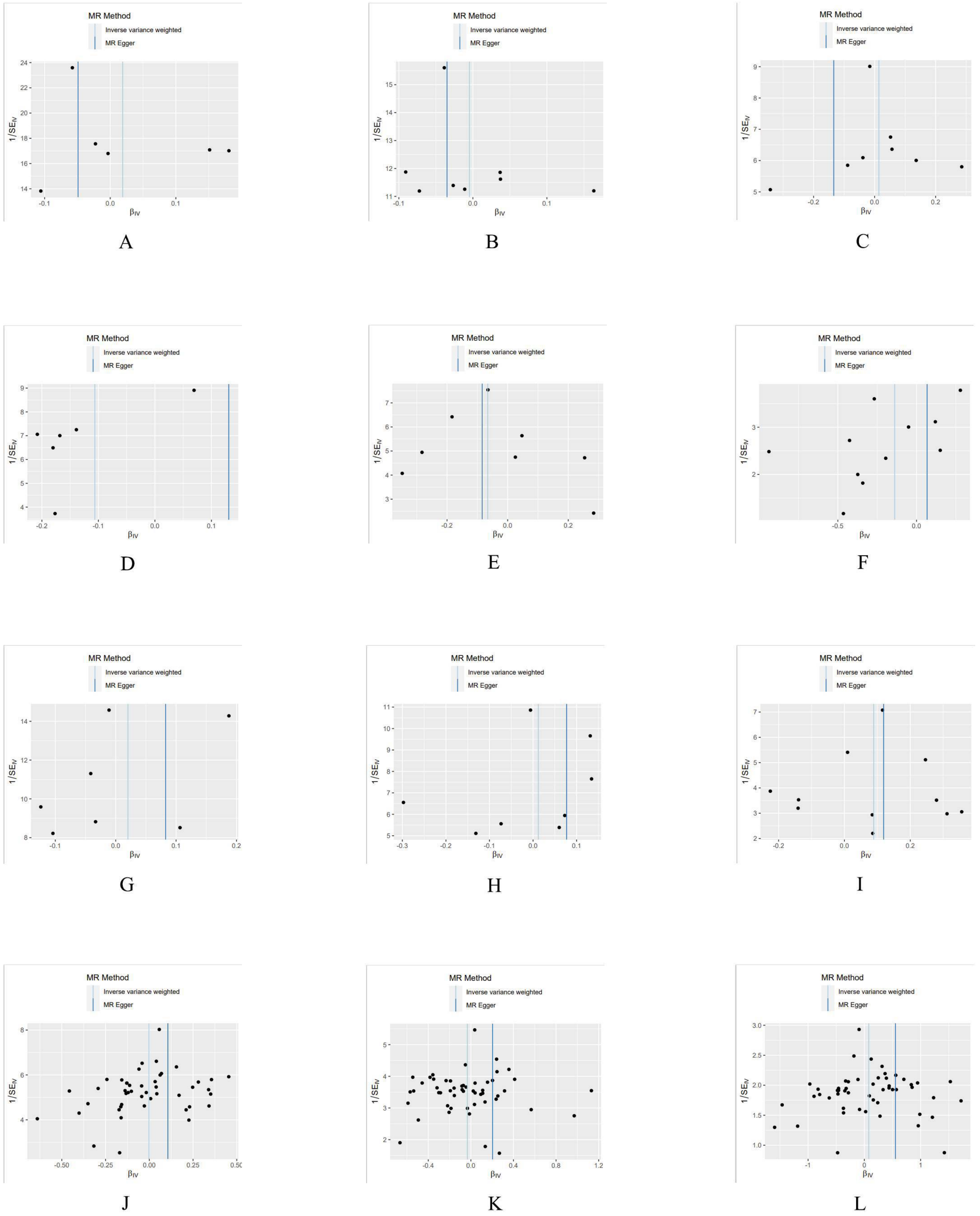

THE FUNNEL PLOT OF THE IMPACT OF BONE FRACTURES AND OSTEOPOROSIS ON THREE PSYCHIATRIC DISORDERS

E.G(EXPOSURE-OUTCOME)

A: (FRACTURE OF NECK-ADHD); B: (FRACTURE OF NECK-ASD);
C: (FRACTURE OF NECK-TS); D: (FRACTURE OF RIB(S), STERNUM AND THORACIC SPINE-ADHD);
E: (FRACTURE OF RIB(S), STERNUM AND THORACIC SPINE-ASD);
F: (FRACTURE OF RIB(S), STERNUM AND THORACIC SPINE-TS);
G: (FRACTURE OF SKULL AND FACIAL BONES-ADHD); H: (FRACTURE OF SKULL AND FACIAL BONES-ASD);
I : (FRACTURE OF SKULL AND FACIAL BONES-TS); G: (FRACTURE OF FOREARM-ADHD);
K: (FRACTURE OF FOREARM-ASD); L: (FRACTURE OF FOREARM-TS)

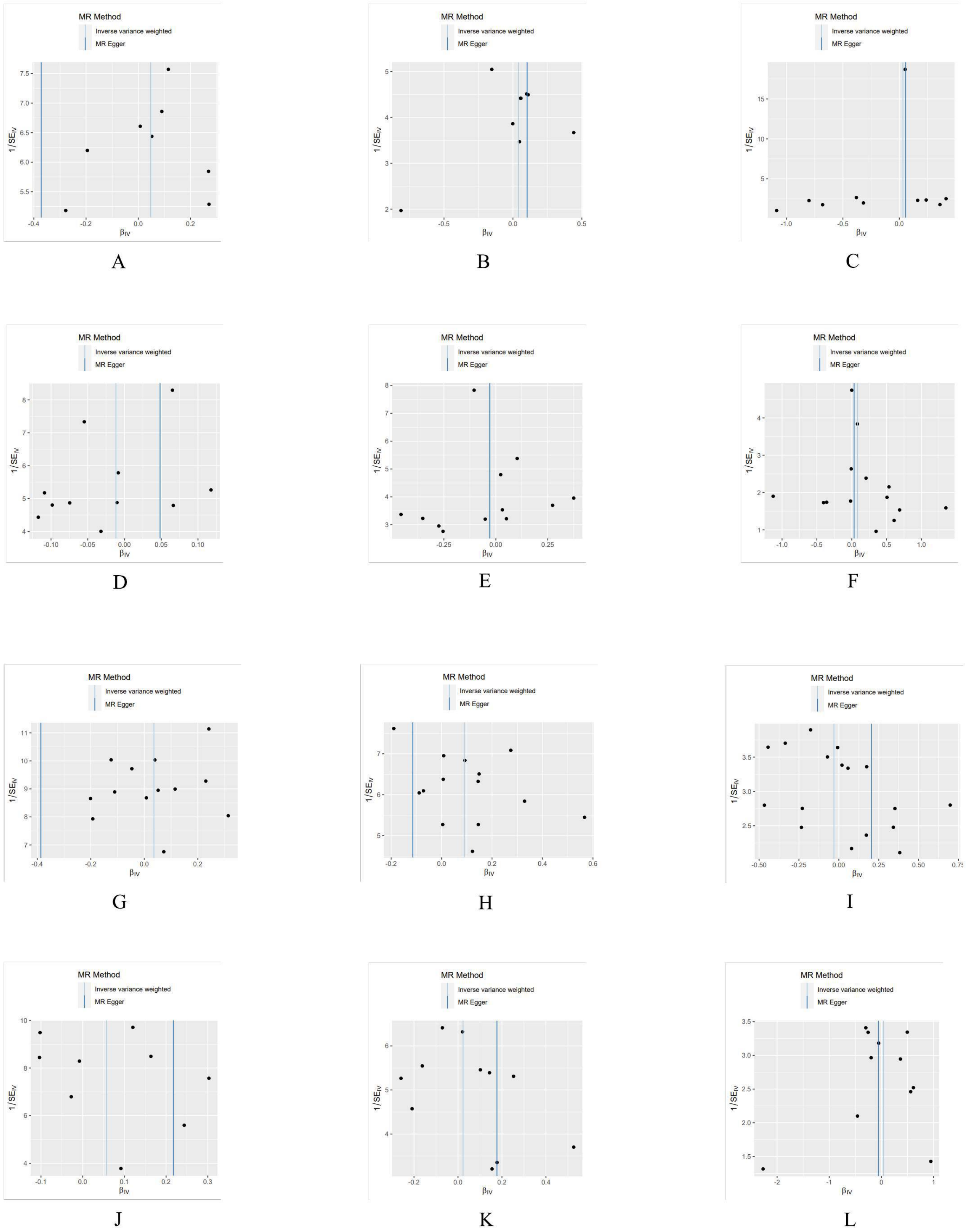

THE FUNNEL PLOT OF THE IMPACT OF BONE FRACTURES AND OSTEOPOROSIS ON THREE PSYCHIATRIC DISORDERS

E.G(EXPOSURE-OUTCOME)

A: (FRACTURE AT WRIST AND HAND LEVEL-ADHD); B: (FRACTURE AT WRIST AND HAND LEVEL-ASD);
C: (FRACTURE AT WRIST AND HAND LEVEL-TS); D: (FRACTURE OF LOWER LEG, INCLUDING ANKLE-ADHD);
E: (FRACTURE OF LOWER LEG, INCLUDING ANKLE-ASD);
F: (FRACTURE OF LOWER LEG, INCLUDING ANKLE-TS);
G: (FRACTURE OF LUMBAR SPINE AND PELVIS-ADHD); H: (FRACTURE OF LUMBAR SPINE AND PELVIS-ASD);
I : (FRACTURE OF LUMBAR SPINE AND PELVIS-TS); G: (FRACTURE OF FOOT, EXCEPT ANKLE-ADHD);
K: (FRACTURE OF FOOT, EXCEPT ANKLE-ASD); L: (FFRACTURE OF FOOT, EXCEPT ANKLE-TS)

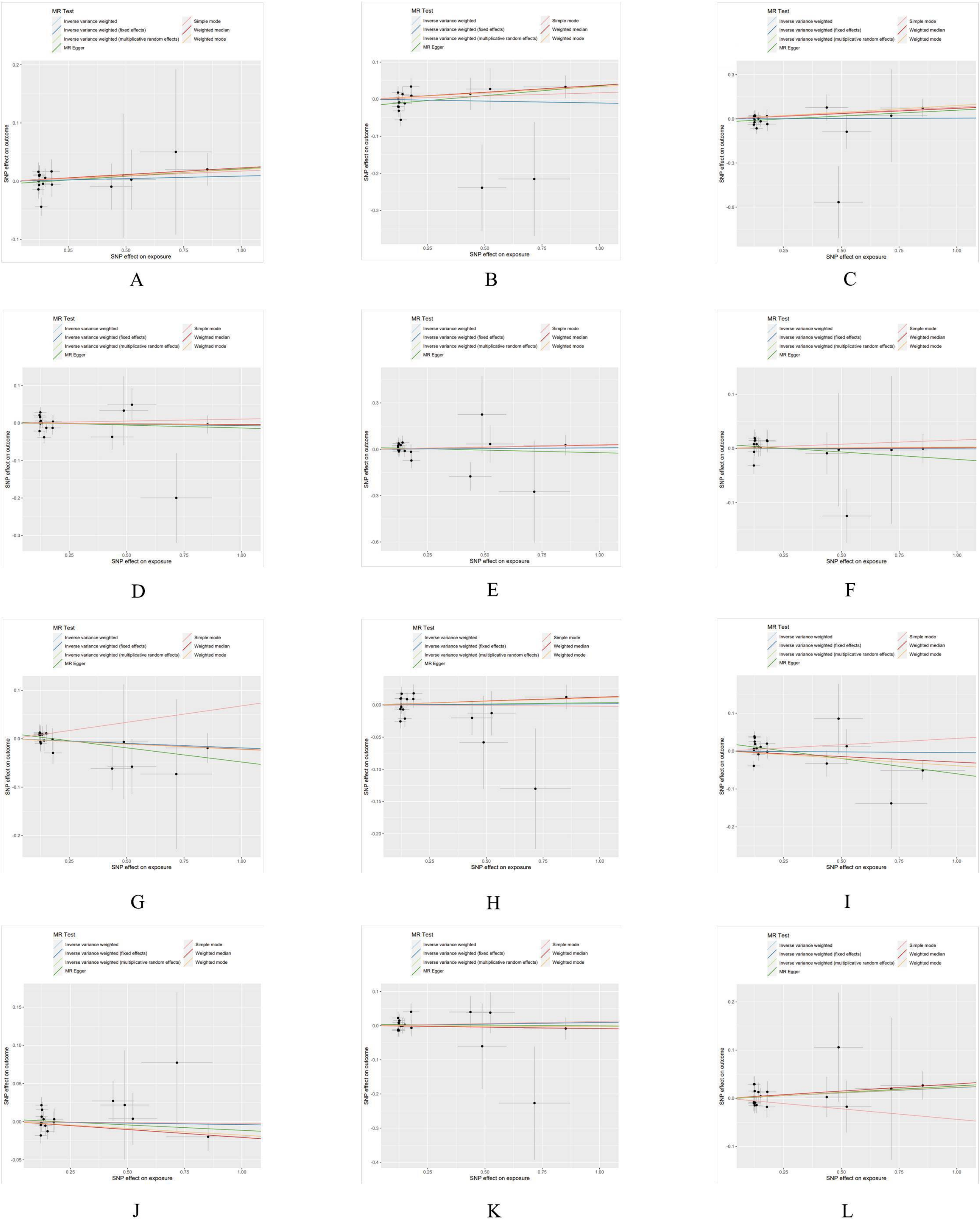

THE SCATTER PLOT OF THE IMPACT OF BONE FRACTURES AND OSTEOPOROSIS ON THREE PSYCHIATRIC DISORDERS

E.G(EXPOSURE-OUTCOME)

A: (FRACTURE OF FEMUR-ADHD); B: (FRACTURE OF FEMUR-ASD);

C: (FRACTURE OF FEMUR-TS); D: (OSTEOPOROSIS-ADHD);E: (OSTEOPOROSIS-ASD);F: (OSTEOPOROSIS-TS);

G: (OSTEOPOROSIS WITH PATHOLOGICAL FRACTURE (FG)-ADHD);

H: (OSTEOPOROSIS WITH PATHOLOGICAL FRACTURE (FG)-ASD);

I: (OSTEOPOROSIS WITH PATHOLOGICAL FRACTURE (FG)-TS);

G: (FRACTURE OF SHOULDER AND UPPER ARM-ADHD); K: (FRACTURE OF SHOULDER AND UPPER ARM-ASD);

L: (FRACTURE OF SHOULDER AND UPPER ARM-TS)

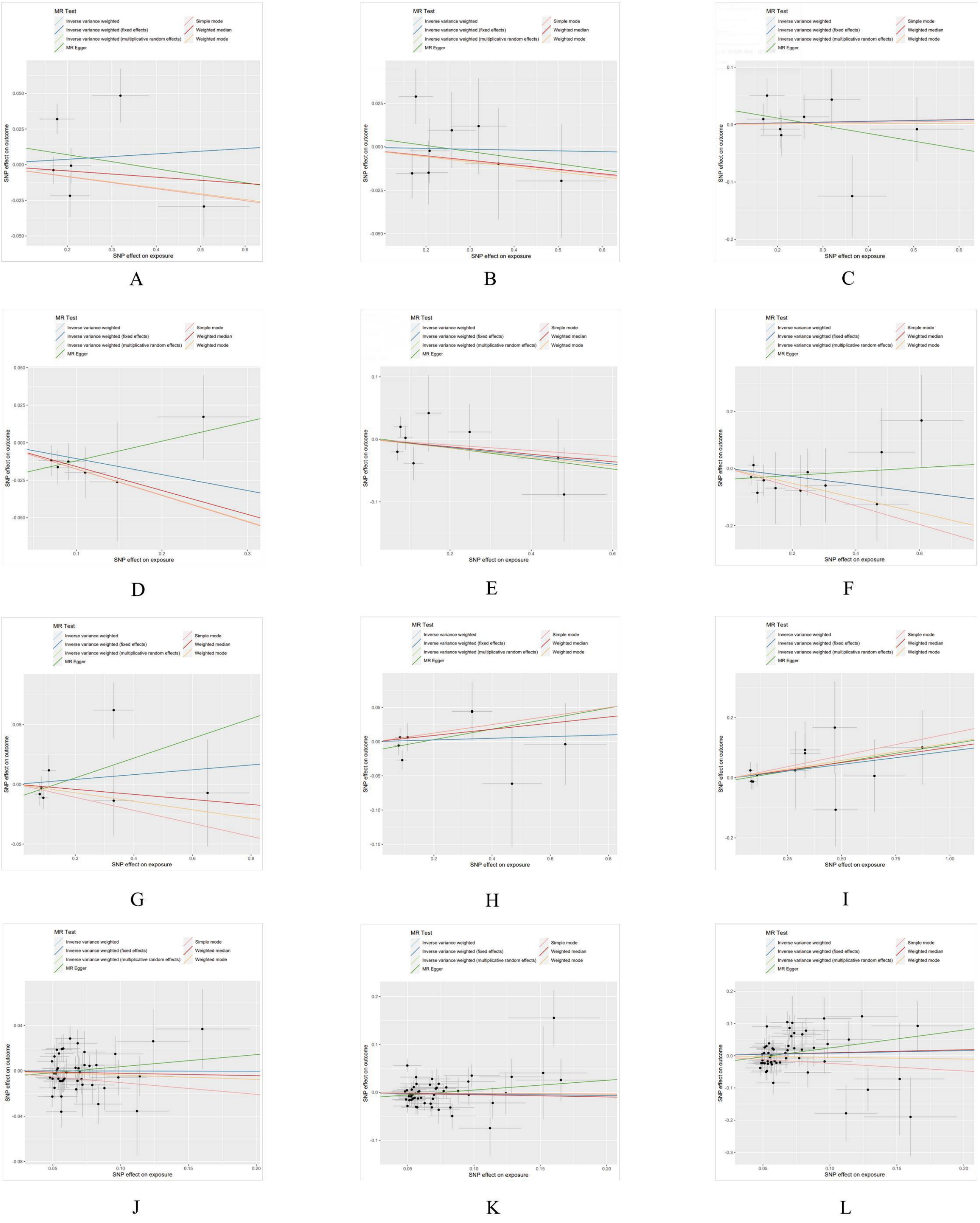

THE SCATTER PLOT OF THE IMPACT OF BONE FRACTURES AND OSTEOPOROSIS ON THREE PSYCHIATRIC DISORDERS

E.G(EXPOSURE-OUTCOME)

A: (FRACTURE OF NECK-ADHD); B: (FRACTURE OF NECK-ASD);
C: (FRACTURE OF NECK-TS); D: (FRACTURE OF RIB(S), STERNUM AND THORACIC SPINE-ADHD);
E: (FRACTURE OF RIB(S), STERNUM AND THORACIC SPINE-ASD);
F: (FRACTURE OF RIB(S), STERNUM AND THORACIC SPINE-TS);
G: (FRACTURE OF SKULL AND FACIAL BONES-ADHD); H: (FRACTURE OF SKULL AND FACIAL BONES-ASD);
I : (FRACTURE OF SKULL AND FACIAL BONES-TS); G: (FRACTURE OF FOREARM-ADHD);
K: (FRACTURE OF FOREARM-ASD); L: (FRACTURE OF FOREARM-TS)

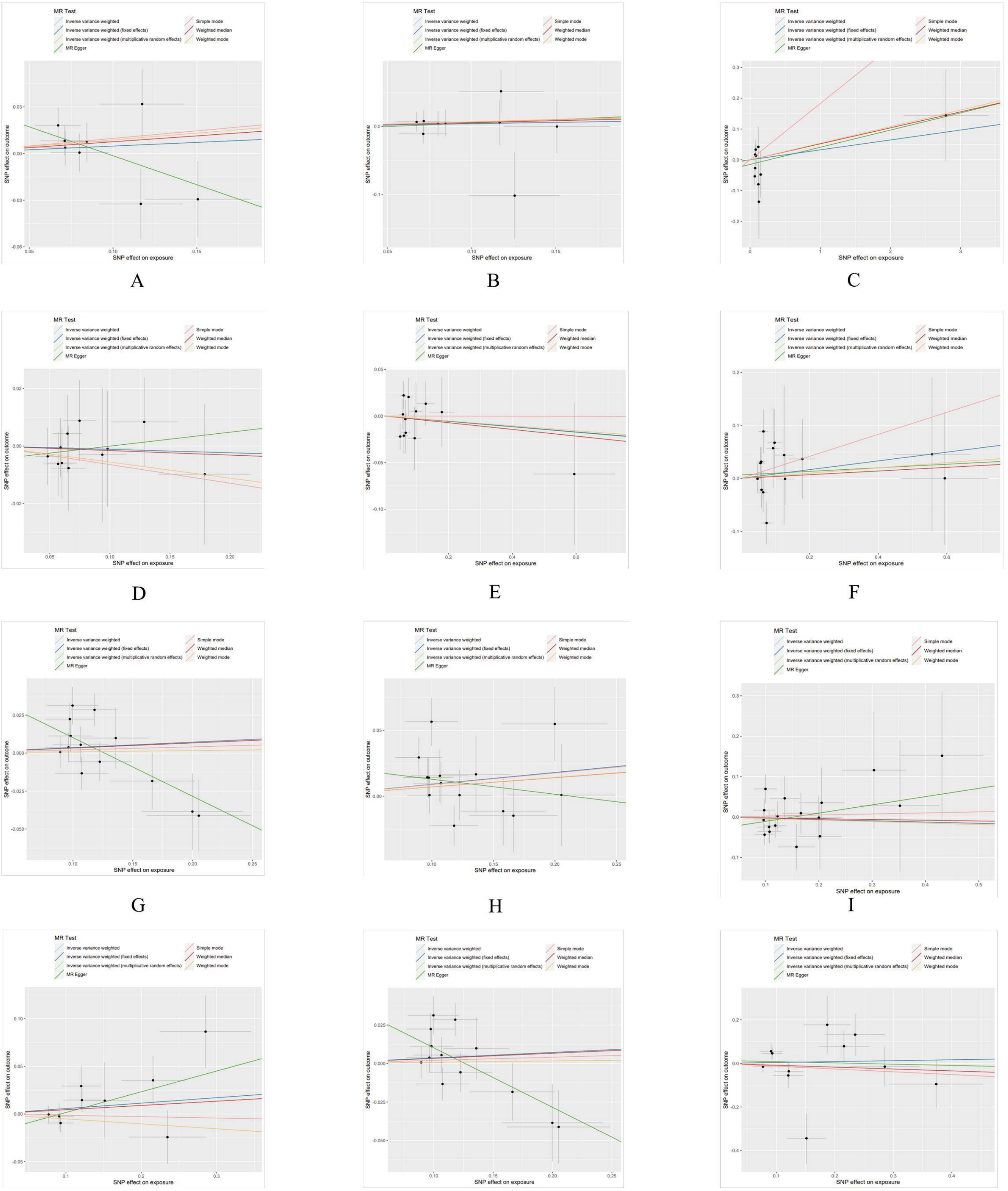

THE SCATTER PLOT OF THE IMPACT OF BONE FRACTURES AND OSTEOPOROSIS ON THREE PSYCHIATRIC DISORDERS

E.G(EXPOSURE-OUTCOME)

A: (FRACTURE AT WRIST AND HAND LEVEL-ADHD); B: (FRACTURE AT WRIST AND HAND LEVEL-ASD);
C: (FRACTURE AT WRIST AND HAND LEVEL-TS); D: (FRACTURE OF LOWER LEG, INCLUDING ANKLE-ADHD);
E: (FRACTURE OF LOWER LEG, INCLUDING ANKLE-ASD);
F: (FRACTURE OF LOWER LEG, INCLUDING ANKLE-TS);
G: (FRACTURE OF LUMBAR SPINE AND PELVIS-ADHD); H: (FRACTURE OF LUMBAR SPINE AND PELVIS-ASD);
I : (FRACTURE OF LUMBAR SPINE AND PELVIS-TS); G: (FRACTURE OF FOOT, EXCEPT ANKLE-ADHD);
K: (FRACTURE OF FOOT, EXCEPT ANKLE-ASD); L: (FFRACTURE OF FOOT, EXCEPT ANKLE-TS)
